# Supplementary material for: Quantum speedup in the identification of cause–effect relations
Source: Nat Commun. 2019 Apr 1;10:1472. doi: 10.1038/s41467-019-09383-8 (PMC6443664; doi:10.1038/s41467-019-09383-8)
Supplement: Supplementary file 1 — Supplementary Information [file 41467_2019_9383_MOESM1_ESM.pdf]

Quantum speedup in the identification of cause-effect relations: supplemental material

Giulio Chiribella*[1,2,3] and Daniel Ebler[4,1]

[1] *Department of Computer Science, The University of Hong Kong, Pokfulam Road, Hong Kong**

[2] *Department of Computer Science, University of Oxford, Oxford, OX1 3QD, United Kingdom*

[3] *Perimeter Institute for Theoretical Physics, Waterloo, Ontario N2L 2Y5, Canada and*

[4] *Institute for Quantum Science and Engineering, Department of Physics, Southern University of Science and Technology, Shenzhen, China.*

* giulio@cs.hku.hk

SUPPLEMENTARY NOTES

Supplementary Note 1: Complementarity relation between tests of the causal structure and tests of the functional dependency between cause and effect.

Here we provide the proof of the complementarity relation (7) in the main text.

1. *Bound on the error probability for parallel strategies with no reference system*

The two causal hypotheses are that the quantum channel from A to the composite system $B \otimes C$ is either of the form $\mathcal{C}_{1,U_1} = \mathcal{U}_{1,B} \otimes I_C/d$, or of the form $\mathcal{C}_{2,U_2} = I_B/d \otimes \mathcal{U}_{2,C}$, with $\mathcal{U}_1(\cdot) := U_1 \cdot U_1^\dagger$, $\mathcal{U}_2(\cdot) := U_2 \cdot U_2^\dagger$. Here, U_1 and U_2 are unitary operations, unknown to the experimenter but fixed throughout the N rounds of the experiment.

Here we consider parallel strategies, where the channel $\mathcal{C}_{x,U_x}^{\otimes N}$ (with $x = 1$ or $x = 2$) is applied in parallel on a multipartite input state, as in the following diagram

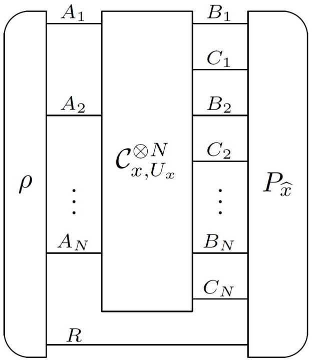

, (1)

where R is a reference system of fixed dimension.

The probability to obtain the outcome $\widehat{x}$ when the channel is $\mathcal{C}_{x,U_x}$ is equal to

$$p(\widehat{x}|x) = \mathrm{Tr}\left[P_{\widehat{x}}\left(\mathcal{C}_{x,U_x}^{\otimes N} \otimes \mathcal{I}_R\right)(\rho)\right]. \tag{2}$$

For fixed gates U_1 and U_2, the probability of error is

$$p_{\mathrm{err}}(U_1, U_2) = \frac{1}{2}\,\mathrm{Tr}\left[P_1\left(\mathcal{C}_{2,U_2}^{\otimes N} \otimes \mathcal{I}_R\right)(\rho)\right] + \frac{1}{2}\,\mathrm{Tr}\left[P_2\left(\mathcal{C}_{1,U_1}^{\otimes N} \otimes \mathcal{I}_R\right)(\rho)\right]. \tag{3}$$

Since U_1 and U_2 are unknown, we consider the worst-case error probability, namely

$$p_{\mathrm{err}}^{\mathrm{wc}} := \max_{U_1, U_2 \in \mathsf{U}} p_{\mathrm{err}}(U_1, U_2), \tag{4}$$

where U is a set of unitary operators. For example, U can be

1. the group of permutation operators of the form $U_\pi = \sum_{i=1}^d |\pi(i)\rangle\langle i|$, where π is an element of the permutation group S_d
2. the group of all unitary operators in dimension d.

In general, we assume that the set U is a generalised N-design [1], meaning that (i) U is a subset of a group representation $\{U_g\}_{g \in \mathsf{G}}$ for some group G, and (ii) for every operator A, one has the identity

$$\frac{1}{|\mathsf{U}|} \sum_{U \in \mathsf{U}} U^{\otimes N} A U^{\otimes N \dagger} = \int_{\mathsf{G}} \mathrm{d}g\, U_g^{\otimes N} A U_g^{\otimes N \dagger}, \tag{5}$$

where $\mathrm{d}g$ denotes the normalized invariant measure over G (for finite groups, it is understood that the integral $\int_{\mathsf{G}} \mathrm{d}g$ has to be replaced by the sum $\frac{1}{|\mathsf{G}|}\sum_g$).

The worst-case error probability is lower bounded by the average error probability

$$p_{\rm err}^{\rm ave} := \frac{1}{|\mathsf{U}|^2} \sum_{U_1,U_2} p_{\rm err}(U_1,U_2)\,. \qquad (6)$$

By definition, the average error probability is equal to the error probability in distinguishing between the *average channels*

$$\mathcal{C}_1^{(N)} := \frac{1}{|\mathsf{U}|} \sum_{U_1} \mathcal{C}_{1,U_1}^{\otimes N} \qquad \text{and} \qquad \mathcal{C}_2^{(N)} := \frac{1}{|\mathsf{U}|} \sum_{U_2} \mathcal{C}_{2,U_2}^{\otimes N}\,. \qquad (7)$$

Now, suppose that the experimenter prepares an N-particle state $|\Psi\rangle \in \mathcal{H}^{\otimes N}$, without using a reference system. The average error probability has the tight lower bound

$$p_{\rm err}^{\rm ave} \ge \frac{1-\frac{1}{2}\left\|\mathcal{C}_1^{(N)}(\Psi)-\mathcal{C}_2^{(N)}(\Psi)\right\|_1}{2} \qquad (8)$$

achieved by Helstrom's minimum error measurement [2]. The distance between the average output states can be expressed as

$$\begin{aligned}
\left\|\mathcal{C}_1^{(N)}(\Psi)-\mathcal{C}_2^{(N)}(\Psi)\right\|_1 &= \left\|\langle\Psi\rangle\otimes\left(\frac{I}{d}\right)^{\otimes N}-\left(\frac{I}{d}\right)^{\otimes N}\otimes\langle\Psi\rangle\right\|_1 && \langle\Psi\rangle := \frac{1}{|\mathsf{U}|}\sum_{U\in\mathsf{U}} U^{\otimes N}\Psi U^{\dagger\,\otimes N}\\
&= \frac{1}{d^N}\sum_{i,j=0}^{d^N-1} |p_i-p_j| && \langle\Psi\rangle = \sum_{i=0}^{d^N-1} p_i\,|i\rangle\langle i|\\
&= \frac{1}{d^N}\sum_{k=1}^{d^N-1} \left\|\langle\Psi\rangle - S^k\langle\Psi\rangle S^{k\dagger}\right\|_1 && S := \sum_{i=0}^{d^N-1} |(i+1) \bmod d^N\rangle\langle i|\\
&= \left(1-\frac{1}{d^N}\right)\|\langle\Psi\rangle\otimes\omega-\Sigma\|_1\,, && (9)
\end{aligned}$$

with

$$\omega := \frac{\sum_{k=1}^{d^N-1}|k\rangle\langle k|}{d^N-1} \qquad \text{and} \qquad \Sigma := \frac{1}{d^N-1}\sum_{k=1}^{d^N-1} S^k\langle\Psi\rangle S^{k\dagger}\otimes|k\rangle\langle k|\,. \qquad (10)$$

Now, the pure states

$$\begin{aligned}
|\Gamma\rangle &:= \sum_{i=0}^{d^N-1}\sum_{k=1}^{d^N-1}\sqrt{\frac{p_i}{d^N-1}}\,|i\rangle\otimes|i\rangle\otimes|k\rangle\otimes|k\rangle\\
|\Delta\rangle &:= \sum_{j=0}^{d^N-1}\sum_{l=1}^{d^N-1}\sqrt{\frac{p_i}{d^N-1}}\,S^k|j\rangle\otimes S^k|j\rangle\otimes|l\rangle\otimes|l\rangle \qquad (11)
\end{aligned}$$

are purifications of $\langle\Psi\rangle\otimes\omega$ and Σ, respectively. Hence, the monotonicity of the trace distance yields the bound

$$\begin{aligned}
\left\|\mathcal{C}_1^{(N)}(\Psi)-\mathcal{C}_2^{(N)}(\Psi)\right\|_1 &\le \left(1-\frac{1}{d^N}\right)\|\Gamma-\Delta\|_1\\
&= \left(1-\frac{1}{d^N}\right)2\sqrt{1-|\langle\Gamma|\Delta\rangle|^2}\\
&\le \left(1-\frac{1}{d^N}\right)2\left(1-\frac{|\langle\Gamma|\Delta\rangle|^2}{2}\right)\,. \qquad (12)
\end{aligned}$$

Inserting this bound into Equation (8), we then obtain

$$p_{\rm err}^{\rm ave} \ge \frac{1}{2d^N}\left[1+\left(d^N-1\right)\frac{|\langle\Gamma|\Delta\rangle|^2}{2}\right] \qquad (13)$$

Now, note that we have

$$
\begin{aligned}
\langle\Gamma|\Delta\rangle &= \frac{1}{d^N-1}\sum_{i,j=0}^{d^N-1}\sqrt{p_ip_j}\;\langle i|\left(\sum_{k=1}^{d^N-1}S^k|j\rangle\langle j|S^k\right)|i\rangle \\
&= \frac{1}{d^N-1}\sum_{i,j=0}^{d^N-1}\sqrt{p_ip_j}\;\langle i|\Big(I-|j\rangle\langle j|\Big)|i\rangle \\
&= \frac{1}{d^N-1}\sum_{i,j=0}^{d^N-1}\sqrt{p_ip_j}\;(1-\delta_{ij}) \\
&= \frac{\left(\mathrm{Tr}\left[\sqrt{\langle\Psi\rangle}\right]\right)^2-1}{d^N-1}
\end{aligned}
\tag{14}
$$

Hence, the Equation (13) yields the bound

$$
p_{\rm err}^{\rm wc}\ge p_{\rm err}^{\rm ave}\ge\frac{1}{2d^N}\left\{1+\frac{\left[\left(\mathrm{Tr}\left[\sqrt{\langle\Psi\rangle}\right]\right)^2-1\right]^2}{2(d^N-1)}\right\}. \tag{15}
$$

It is clear that the minimum of the right-hand-side is obtained when the state $\langle\Psi\rangle$ is pure, in which case, the bound becomes $p_{\rm err}^{\rm wc}\ge 1/(2d^N)$.

2. Bound on the success probability in the identification of a unitary gate

More generally, the bound (15) can be interpreted as a complementarity relation between the estimation of the causal structure and the estimation of the functional dependence between cause and effect.

Lemma 1. Consider the task of guessing the gate $U\in\mathsf{U}$ from the state $|\Psi_U\rangle:=U^{\otimes N}|\Psi\rangle$. If U is a generalised N-design for some group representation $\{U\}_{U\in\mathsf{G}}$, then the probability of a correct guess satisfies the bound

$$
p_{\rm guess}^{\mathsf{U}}\le\frac{\left(\mathrm{Tr}\left[\sqrt{\langle\Psi\rangle}\right]\right)^2}{|\mathsf{U}|} \tag{16}
$$

The bound is attained by the square-root measurement [3], with operators $P_U=\langle\Psi\rangle^{-\frac12}\,\Psi_U\langle\Psi\rangle^{-\frac12}/|\mathsf{U}|$.

Proof. Equation (16) follows from the Yuen-Kennedy-Lax bound [4] $p_{\rm guess}^{\mathsf{U}}\le\mathrm{Tr}[\Lambda]$ where Λ is a positive operator satisfying the inequalities $\Lambda\ge\frac{1}{|\mathsf{U}|}U^{\otimes N}\Psi U^{\otimes N\dagger}$ for all $U\in\mathsf{U}$. Equivalently, one has $U^{\otimes N\dagger}\Lambda U^{\otimes N}\le\Psi/|\mathsf{U}|$ for all U, which implies the condition

$$
\langle\Lambda\rangle\ge\frac{\Psi}{|\mathsf{U}|},\qquad\langle\Lambda\rangle:=\frac{1}{|\mathsf{U}|}\sum_U U^{\otimes N\dagger}\Lambda U^{\otimes N}. \tag{17}
$$

Then, the Yuen-Kennedy-Lax bound implies the inequality

$$
p_{\rm guess}^{\mathsf{U}}\le\mathrm{Tr}[\langle\Lambda\rangle]. \tag{18}
$$

Since the unitaries U form a generalised N-design, the operator $\langle\Lambda\rangle$ is invariant under the action of the group representation $\{U^{\otimes N}\}_{U\in\mathsf{G}}$. Moreover, every invariant operator Γ can be written as $\langle\Lambda\rangle$ for some suitable Λ (in fact, it suffices to take $\Lambda=\Gamma$). Hence, one has the bound

$$
p_{\rm guess}^{\mathsf{U}}\le\mathrm{Tr}[\Gamma],\qquad\forall\Gamma:\,\langle\Gamma\rangle=\Gamma,\quad\Gamma\ge\frac{\Psi}{|\mathsf{U}|}. \tag{19}
$$

In particular, one can take $\Gamma = c\sqrt{\langle\Psi\rangle}$ for some suitable constant c. With this choice, the condition $\Gamma \geq \Psi/|\mathsf{U}|$ is equivalent to

$$c\, I \geq \frac{\langle\Psi\rangle^{-\frac{1}{4}}\,\Psi\,\langle\Psi\rangle^{-\frac{1}{4}}}{|\mathsf{U}|}\,, \tag{20}$$

which in turn is equivalent to

$$c \geq \frac{\mathrm{Tr}[\Psi\langle\Psi\rangle^{-\frac{1}{2}}]}{|\mathsf{U}|} = \frac{\mathrm{Tr}[\langle\Psi\rangle\langle\Psi\rangle^{-\frac{1}{2}}]}{|\mathsf{U}|} = \frac{\mathrm{Tr}[\langle\Psi\rangle^{\frac{1}{2}}]}{|\mathsf{U}|}\,. \tag{21}$$

Then, the bound (19) becomes $p_{\mathrm{guess}}^{\mathsf{U}} \leq \mathrm{Tr}[\sqrt{\langle\Psi\rangle}]^2/|\mathsf{U}|$. The bound is attained by the square-root measurement $P_U = \langle\Psi\rangle^{-\frac{1}{2}}\,\Psi_U\,\langle\Psi\rangle^{-\frac{1}{2}}/|\mathsf{U}|$, which yields

$$\begin{aligned}
\mathrm{Tr}[P_U\,\Psi_U] &= \frac{1}{|\mathsf{U}|}\,\mathrm{Tr}[\langle\Psi\rangle^{-\frac{1}{2}}\,\Psi_U\,\langle\Psi\rangle^{-\frac{1}{2}}\,\Psi_U] \\
&= \frac{1}{|\mathsf{U}|}\,\mathrm{Tr}[\langle\Psi\rangle^{-\frac{1}{2}}\,\Psi\langle\Psi\rangle^{-\frac{1}{2}}\Psi] \\
&= \frac{1}{|\mathsf{U}|}\,\left|\langle\Psi|\langle\Psi\rangle^{-\frac{1}{2}}|\Psi\rangle\right|^2 \\
&= \frac{1}{|\mathsf{U}|}\,\left|\,\mathrm{Tr}[\Psi\langle\Psi\rangle^{-\frac{1}{2}}]\right|^2 \\
&= \frac{1}{|\mathsf{U}|}\,\left|\,\mathrm{Tr}[\langle\Psi\rangle\langle\Psi\rangle^{-\frac{1}{2}}]\right|^2 \\
&= \frac{1}{|\mathsf{U}|}\,\left|\,\mathrm{Tr}[\langle\Psi\rangle^{\frac{1}{2}}]\right|^2,
\end{aligned} \tag{22}$$

for every U. ☐

Combining the above lemma with Equation (15) we obtain the relation

$$p_{\mathrm{err}}^{\mathrm{wc}} \geq \frac{1}{2d^N}\left\{1 + \frac{1}{2(d^N-1)}\left(\frac{p_{\mathrm{guess}}^{\mathsf{U}} - \frac{1}{|\mathsf{U}|}}{\frac{1}{|\mathsf{U}|}}\right)^2\right\}\,. \tag{23}$$

Supplementary Note 2: Optimal universal strategy

Here we derive the optimal strategy for identifying the causal intermediary when the cause-effect relationship is described by an arbitrary unitary gate.

3. Reduction to the minimisation of the average probability

The problem is to find the strategy that minimises the worst-case error probability. Thanks to the symmetry of the problem, the minimisation of the worst-case error probability can be reduced to the minimisation of the average error probability:

Lemma 2. For every fixed reference system R and for every fixed N, minimum worst-case error probability in the discrimination of the channels $\mathcal{C}_{1,U_1}$ and $\mathcal{C}_{2,U_2}$ with N uses is equal to the average error probability

$$p_{\mathrm{err}}^{\mathrm{ave}} := \int_{U_1\in\mathsf{SU}(d)} \mathrm{d}U_1 \int_{U_2\in\mathsf{SU}(d)} \mathrm{d}U_2\; p_{\mathrm{err}}(U_1,U_2)\,, \tag{24}$$

where $\mathrm{d}U$ is the normalised invariant measure. In turn, the average error probability is equal to the minimum error probability in the discrimination of the channels

$$\mathcal{C}_1^{(N)} := \int \mathrm{d}U_1\; \mathcal{C}_{1,U_1}^{\otimes N} \qquad \text{and} \qquad \mathcal{C}_2^{(N)} := \int \mathrm{d}U_2\; \mathcal{C}_{2,U_2}^{\otimes N}\,. \tag{25}$$

There exists a state ρ and a measurement $\{P_1, P_2\}$ that are optimal for both problems.

We omit the proof, which is a simple adaptation of Holevo's argument on the optimality of covariant measurements [5], see also [6].

4. Optimal form of the input states

Let us search for the optimal quantum strategy. Note that the channels $\mathcal{C}_1^{(N)}$ and $\mathcal{C}_2^{(N)}$ satisfy the condition

$$\mathcal{C}_x^{(N)} = \mathcal{C}_x^{(N)} \circ \mathcal{T}_{\rm in}^{(N)} \,, \qquad \forall x \in \{1,2\} \,. \tag{26}$$

where $\mathcal{T}_{\rm in}^{(N)}$ is the twirling channel

$$\mathcal{T}_{\rm in}^{(N)} := \int {\rm d}W \, \mathcal{W}^{\otimes N} \,. \tag{27}$$

Eq. (26) implies that the search of the optimal input state can be restricted to invariant states—*i. e.* states satisfying the condition

$$\mathcal{T}_{\rm in}^{(N)}(\rho) = \rho \,. \tag{28}$$

The structure of the invariant states can be made explicit using the Schur-Weyl duality [7], whereby the tensor product Hilbert space $\mathcal{H}^{\otimes N}$ is decomposed as

$$\mathcal{H}^{\otimes N} = \bigoplus_{\lambda \in \mathsf{Y}_{N,d}} \left(\mathcal{R}_\lambda \otimes \mathcal{M}_\lambda \right) , \tag{29}$$

where $\mathsf{Y}_{N,d}$ is the set of Young diagrams of N boxes arranged in d rows, while $\mathcal{R}_\lambda$ and $\mathcal{M}_\lambda$ are representation and multiplicity spaces for the tensor action of $\mathsf{SU}(d)$, respectively. Using the Schur-Weyl decomposition, every invariant state on $\mathcal{H}^{\otimes N} \otimes \mathcal{H}_R$ can be decomposed as

$$\rho = \bigoplus_\lambda q_\lambda \left(\frac{P_\lambda}{d_\lambda} \otimes \rho_{\lambda R} \right) , \tag{30}$$

where $\{q_\lambda\}$ is a probability distribution, P_λ is the identity operator on the representation space $\mathcal{R}_\lambda$, and $\rho_{\lambda R}$ is a density matrix on the Hilbert space $\mathcal{M}_\lambda \otimes \mathcal{H}_R$.

Note that the set of invariant states (30) is convex. Since the (average) error probability is a linear function of ρ, the minimisation can be restricted to the extreme points of this convex set. Hence, we have the following

Proposition 1. Without loss of generality, the optimal input state for a parallel strategy with reference system R can be taken of the form

$$\rho = \frac{P_{\lambda_0}}{d_{\lambda_0}} \otimes \Psi_{\lambda_0 R} \,, \tag{31}$$

where $\lambda_0 \in \mathsf{Y}_{N,d}$ is a fixed Young diagram and $\Psi_{\lambda_0 R}$ is a pure state on $\mathcal{M}_{\lambda_0} \otimes \mathcal{H}_R$.

5. Error probability for states of the optimal form

The problem is to find the input state that makes the output states most distinguishable. To this purpose, it is convenient to label operators with the corresponding systems and to use the notation $\mathbf{A} := A_1 A_2 \cdots A_N$, $\mathbf{B} := B_1 B_2 \cdots B_N$, $\mathbf{C} := C_1 C_2 \cdots C_N$, and $\mathbf{R} := R$.

When applied to an invariant state of the composite system $\mathbf{AR}$, the two channels $\mathcal{C}_1^{(N)}$ and $\mathcal{C}_2^{(N)}$ produce the output states

$$\left(\mathcal{C}_1^{(N)} \otimes \mathcal{I}_{\mathbf{R}} \right)(\rho_{\mathbf{AR}}) = \rho_{\mathbf{BR}} \otimes \left(\frac{I}{d} \right)_{\mathbf{C}}^{\otimes N} \qquad \text{and} \qquad \left(\mathcal{C}_2^{(N)} \otimes \mathcal{I}_{\mathbf{R}} \right)(\rho_{\mathbf{AR}}) = \left(\frac{I}{d} \right)_{\mathbf{B}}^{\otimes N} \otimes \rho_{\mathbf{CR}} \,, \tag{32}$$

up to a convenient reordering of the Hilbert spaces.

The minimum error probability in the discrimination of the output states is given by Helstrom's theorem [2]. Specifically, one has

$$p_{\mathrm{err}} = \frac{1}{2}\left(1 - \frac{1}{2}\|\Delta\|_1\right), \qquad \Delta := \rho_{\mathbf{BR}} \otimes \left(\frac{I}{d}\right)^{\otimes N}_{\mathbf{C}} - \left(\frac{I}{d}\right)^{\otimes N}_{\mathbf{B}} \otimes \rho_{\mathbf{CR}}\,. \tag{33}$$

In the following, we compute the trace norm explicitly for input states of the optimal form

$$\rho = \frac{P_{\lambda_0}}{d_{\lambda_0}} \otimes \Psi_{\lambda_0 R}\,, \tag{34}$$

It is convenient to decompose the identity operator $I^{\otimes N}$ as

$$I^{\otimes N} = \bigoplus_{\lambda \in \mathsf{Y}_{N,d}} \Big(P_\lambda \otimes Q_\lambda\Big)\,, \tag{35}$$

where P_λ is the identity operator on the representation space $\mathcal{R}_\lambda$ and Q_λ is the identity operator on the multiplicity space $\mathcal{M}_\lambda$. In the following, we denote by $m_\lambda = \mathrm{Tr}[Q_\lambda]$ the dimension of $\mathcal{M}_\lambda$. Combining Eqs. (32), (34), and (35), we obtain

$$\begin{aligned}
\|\Delta\|_1 &= \frac{d_{\lambda_0} m_{\lambda_0}}{d^N} \left\| \frac{P_{\lambda_0}}{d_{\lambda_0}} \otimes \frac{P_{\lambda_0}}{d_{\lambda_0}} \otimes \left(\Psi_{\lambda_0 R} \otimes \frac{Q_{\lambda_0}}{m_{\lambda_0}} - \frac{Q_{\lambda_0}}{m_{\lambda_0}} \otimes \Psi_{\lambda_0 R}\right)\right\|_1 \\
&\quad + 2\sum_{\lambda \neq \lambda_0} \frac{d_\lambda m_\lambda}{d^N} \left\| \frac{P_{\lambda_0}}{d_{\lambda_0}} \otimes \frac{P_\lambda}{d_\lambda} \otimes \Psi_{\lambda_0 R} \otimes \frac{Q_\lambda}{m_\lambda}\right\|_1 \\
&= \frac{d_{\lambda_0} m_{\lambda_0}}{d^N} \left\| \Psi_{\lambda_0 R} \otimes \frac{Q_{\lambda_0}}{m_{\lambda_0}} - \frac{Q_{\lambda_0}}{m_{\lambda_0}} \otimes \Psi_{\lambda_0 R}\right\|_1 + 2\left(1 - \frac{d_{\lambda_0} m_{\lambda_0}}{d^N}\right)
\end{aligned} \tag{36}$$

It remains to compute the trace norm in the first summand. To this purpose, it is convenient to define the states

$$\left|\Phi_n^\pm\right\rangle := \frac{|\Psi_{\lambda_0 R}\rangle \otimes |n\rangle \pm |n\rangle \otimes |\Psi_{\lambda_0 R}\rangle}{\gamma_n^\pm}\,, \qquad \gamma_n^\pm := \sqrt{2(1 \pm \langle n|\rho|n\rangle)}\,, \tag{37}$$

where ρ is the marginal state of $\Psi_{\lambda_0 R}$ on the multiplicity space $\mathcal{M}_{\lambda_0}$, and $\{|n\rangle\,, n = 1, \dots, m_{\lambda_0}\}$ are the eigenvectors of ρ. With this definition, the states

$$\{\left|\Phi_n^k\right\rangle\,, k \in \{+,-\}\,, n \in \{1, \dots, m_{\lambda_0}\}\} \tag{38}$$

are mutually orthogonal. For example, one has

$$\begin{aligned}
\langle \Phi_m^+|\Phi_n^+\rangle &= \frac{\mathrm{Re}[\langle m|\rho|n\rangle]}{\gamma_m^\pm \gamma_n^\pm} \\
&= 0\,,
\end{aligned} \tag{39}$$

the second equality coming from the fact that ρ is diagonal in the basis $\{|n\rangle\}$.

In terms of the vectors (37), one can rewrite the relevant terms as

$$\Psi_{\lambda_0 R} \otimes \frac{Q_{\lambda_0}}{m_{\lambda_0}} - \frac{Q_{\lambda_0}}{m_{\lambda_0}} \otimes \Psi_{\lambda_0 R} = \frac{1}{2m_{\lambda_0}} \sum_n \gamma_n^+ \gamma_n^- \Big(\left|\Phi_n^+\right\rangle \left\langle \Phi_n^-\right| + \left|\Phi_n^-\right\rangle \left\langle \Phi_n^+\right| \Big)\,. \tag{40}$$

Then, the trace norm is

$$\begin{aligned}
\left\| \Psi_{\lambda_0 R} \otimes \frac{Q_{\lambda_0}}{m_{\lambda_0}} - \frac{Q_{\lambda_0}}{m_{\lambda_0}} \otimes \Psi_{\lambda_0 R}\right\|_1 &= \frac{1}{2m_{\lambda_0}} \sum_n \gamma_n^+ \gamma_n^- \left\| \left|\Phi_n^+\right\rangle \left\langle \Phi_n^-\right| + \left|\Phi_n^-\right\rangle \left\langle \Phi_n^+\right| \right\|_1 \\
&= \frac{2}{m_{\lambda_0}} \sum_n \sqrt{1 - \langle n|\rho|n\rangle^2}\,.
\end{aligned} \tag{41}$$

The maximum trace norm is reached when the eigenvalues of ρ are all equal. In that case, one has

$$\left\| \Psi_{\lambda_0 R} \otimes \frac{Q_{\lambda_0}}{m_{\lambda_0}} - \frac{Q_{\lambda_0}}{m_{\lambda_0}} \otimes \Psi_{\lambda_0 R}\right\|_1 = \frac{2}{m_{\lambda_0}} \left(m_{\lambda_0} - r + r\sqrt{1 - r^{-2}}\right), \tag{42}$$

where r is the rank of ρ. Combining the above equation with Eqs. (36) and (33) we obtain the error probability

$$p_{\rm err} = \frac{d_{\lambda_0}}{2d^N}\, f(r) \qquad f(r) := r\left(1-\sqrt{1-r^{-2}}\right) . \tag{43}$$

Note that the function $f(r)$ is monotonically decreasing, and therefore the error probability is minimised by maximising the rank r, *i. e.* by choosing

$$r = \min\{m_{\lambda_0}\,, d_R\}\,, \tag{44}$$

where d_R is the dimension of the reference system.

6. Minimum error probability

The probability of error is given by Eq. (43). When the reference system has dimension larger than the multiplicity m_{λ_0}, one has the equality

$$r = m_{\lambda_0} \tag{45}$$

and the error probability becomes

$$p_{\rm err} = \frac{d_{\lambda_0}}{2d^N}\, f(m_{\lambda_0})\,, \tag{46}$$

with f defined as in Equation (43).

The only way to beat the classical scaling $1/d^N$ is to make $f(m_{\lambda_0})$ exponentially small. Since f is positive and monotonically decreasing, this means that m_{λ_0} must be exponentially large. Note that, for large m_{λ_0}, the probability of error has the asymptotic expression

$$p_{\rm err} = \frac{d_{\lambda_0}}{4m_{\lambda_0}d^N}\,\left[1+O\left({m_{\lambda_0}}^{-2}\right)\right] . \tag{47}$$

Asymptotically, the problem is reduced to the minimisation of the ratio $d_{\lambda_0}/m_{\lambda_0}$.

To find the minimum, it is useful to apply the notion of majorisation Young diagrams. Given two diagrams λ and μ of N boxes arranged in d rows, we say that λ *majorises* μ if

$$\sum_{i=1}^{s}\lambda_i \ge \sum_{i=1}^{s}\mu_i \qquad \forall s\in\{1,\dots,d\}\,, \tag{48}$$

where λ_i (μ_i) is the length of the i-th row of the diagram λ (μ).

Lemma 3. If λ majorises μ, then $d_\lambda/m_\lambda \ge d_\mu/m_\mu$.

Proof. For a generic Young diagram $\lambda\in\mathsf{Y}_{N+1,d}$, one has

$$d_\lambda = \frac{\prod_{(i,j)\in\lambda} d-i+j}{\prod_{(i,j)\in\lambda}{\rm hook}(i,j)} \qquad \text{and} \qquad m_\lambda = \frac{N!}{\prod_{(i,j)\in\lambda}{\rm hook}(i,j)}\ , \tag{49}$$

Here the pair (i,j) labels a box in the diagram, with the indices i and j labelling the row and the column, respectively. ${\rm hook}(i,j)$ denotes the length of the hook consisting of boxes to the right and to the bottom of the box (i,j). Using the above expressions, the dimension/multiplicity ratio reads

$$\begin{aligned}\frac{d_\lambda}{m_\lambda} &= \frac{\prod_{(i,j)\in\lambda} d-i+j}{N!}\\ &= \frac{1}{N!}\prod_{i=1}^{d}\frac{(d-i+\lambda_i)!}{(d-i)!}\ .\end{aligned} \tag{50}$$

Now, since λ majorises μ, one has the bounds

$$\frac{(d-1+\lambda_1)!}{(d-1)!} \geq \frac{(d-1+\mu_1)!}{(d-1)!}\,(d+\mu_1)^{\lambda_1-\mu_1}$$

$$\frac{(d-1+\lambda_1)!}{(d-1)!}\,\frac{(d-2+\lambda_2)!}{(d-2)!} \geq \frac{(d-1+\mu_1)!}{(d-1)!}\,\frac{(d-2+\mu_2)!}{(d-2)!}\,(d-1+\mu_2)^{\lambda_1+\lambda_2-\mu_1-\mu_2}$$

$$\vdots$$

$$\prod_{i=1}^{s}\frac{(d-i+\lambda_i)!}{(d-i)!} \geq \prod_{i=1}^{s}\frac{(d-i+\mu_i)!}{(d-i)!}\,(d-s+1+\mu_s)^{\sum_{i=1}^{s}(\lambda_i-\mu_i)} \qquad \forall s\in\{1,\dots,d\}\,. \tag{51}$$

Choosing $s=d$ and recalling Eq. (50), one finally obtains $d_\lambda/m_\lambda \geq d_\mu/m_\mu$. □

Proposition 2. Define $t := N - d\lfloor N/d\rfloor$. Then, the ratio d_λ/m_λ is

1. minimum when λ is the Young diagram with t rows of length $\lceil N/d\rceil$ and $d-t$ rows of length $\lfloor N/d\rfloor$
2. maximum when λ is the Young diagram with one row or length N.

Proof. The Young diagram $\lambda_0 = (\underbrace{\lceil N/d\rceil,\dots,\lceil N/d\rceil}_{t\ \text{times}},\underbrace{\lfloor N/d\rfloor,\dots,\lfloor N/d\rfloor}_{d-t\ \text{times}})$ is majorised by any other Young diagram in $\mathsf{Y}_{N,d}$. Hence, λ_0 minimises the ratio d_λ/m_λ (by Lemma *3*). Similarly, the Young diagram $\lambda_0 = (N,\underbrace{0,\dots,0}_{d-1\ \text{times}})$ majorises every other young diagram and therefore it maximises the ratio d_λ/m_λ. □

Summarizing, we showed that

1. when N is a multiple of d, the optimal Young diagram corresponds to the trivial representation of $\mathsf{SU}(d)$
2. when N is not a multiple of d, the optimal Young diagram corresponds to the totally antisymmetric representation acting on $N - d\lfloor N/d\rfloor$ particles.
3. asymptotically, the symmetric subspace is the worst possible choice, leading to the classical rate $R_{\mathrm{C}} = \log d$.

In conclusion, we proved the following

Proposition 3. When N is a multiple of d, the optimal input state is $P_{\lambda_0}/d_{\lambda_0}\otimes|\Psi\rangle\langle\Psi|_{\lambda_0\mathbf{R}}$, where λ_0 is he trivial representation of $\mathsf{SU}(d)$ in the N-fold tensor product $U^{\otimes N}$, $d_R \geq m_{\lambda_0}$, and $|\Psi\rangle_{\lambda_0\mathbf{R}} \in \mathcal{M}_{\lambda_0}\otimes\mathcal{H}_R$ is a maximally entangled state.

Since the trivial representation is one-dimensional, the error probability (47) takes the form

$$p_{\text{err}} = \frac{1}{4m_{\lambda_0}d^N}\,\left[1+O\left({m_{\lambda_0}}^{-2}\right)\right]\,. \tag{52}$$

Moreover, the trivial representation of $\mathsf{SU}(d)$ corresponds to the Young diagram with d rows, each of length N/d. Hence, its multiplicity is given by

$$m_{\lambda_0} = \frac{N!}{\prod_{i=1}^{d}\frac{\left(\frac{N}{d}+d-i\right)!}{(d-i)!}}\,. \tag{53}$$

For fixed d, the Stirling approximation yields the expression

$$m_{\lambda_0} = d^N\left[\frac{d^{\frac{d^2}{2}}\,e^{\frac{d(d-1)}{2}}\prod_{i=1}^{d}(d-i)!}{(2\pi)^{\frac{d-1}{2}}\,N^{\frac{d^2-1}{2}}}\right]c(N)\,, \tag{54}$$

where $c(N)$ is a function tending to 1 in the large N limit. Taking the logarithm on both sides, one obtains

$$\log m_{\lambda_0} = N\log d + O(\log N)\,. \tag{55}$$

Inserting this value into the expression of the error probability (47), we obtain the rate

$$\begin{aligned} R &= \lim_{N\to\infty} -\frac{\log p_{\rm err}}{N} \\ &= \lim_{N\to\infty} \frac{\log(4m_{\lambda_0} d^N)}{N} \\ &= 2\log d\,. \end{aligned} \tag{56}$$

7. *Quantum superposition of equivalent setups*

Here we prove that the optimal state can be realized as a coherent superposition of equivalent setups, where the N input variables are divided in groups of d, and all the variables in the same group are initialized in the $\mathsf{SU}(d)$ singlet state.

Proposition 4. For N multiple of d, consider the state

$$|\Psi\rangle_{\mathbf{AR}} = \frac{1}{\sqrt{G_{N,d}}} \sum_i \left(|S\rangle_{\mathbf{A}}^{\otimes N/d}\right)_i \otimes |i\rangle_{\mathbf{R}}\,, \tag{57}$$

where $\{|i\rangle_{\mathbf{R}}\}_{i=1}^{G_{N,d}}$ is an orthonormal basis for the reference system, indexed by the possible ways to group N objects into groups of d, and $\left(|S\rangle^{\otimes N/d}\right)_i$ is the product of N/d singlet states, distributed according to the grouping i. Then,

1. the state $|\Psi\rangle_{\mathbf{AR}}$ is optimal for the identification of the causal intermediary
2. the number r of linearly independent vectors of the form $\left(|S\rangle_{\mathbf{A}}^{\otimes N/d}\right)_i$ satisfies the equality

$$r = d^N \left[\frac{d^{\frac{d^2}{2}}\, e^{\frac{d(d-1)}{2}} \prod_{i=1}^d (d-i)!}{(2\pi)^{\frac{d-1}{2}}\, N^{\frac{d^2-1}{2}}}\right] c(N)\,, \tag{58}$$

   where $c(N)$ is a function tending to 1 in the large N limit.

Proof. By definition, $|\Psi\rangle_{\mathbf{AR}}$ is invariant under the n-fold action of $\mathsf{SU}(d)$ on system $\mathbf{A}$, meaning that the corresponding density matrix has the optimal form $|\Psi\rangle\langle\Psi|_{\mathbf{AR}} = P_{\lambda_0}/d_{\lambda_0} \otimes |\Psi\rangle\langle\Psi|_{\lambda_0 \mathbf{R}}$, where λ_0 is the trivial representation of $\mathsf{SU}(d)$. In fact, since the trivial representation is one-dimensional, we may equivalently write $|\Psi\rangle\langle\Psi|_{\mathbf{AR}} \equiv |\Psi\rangle\langle\Psi|_{\lambda_0\mathbf{R}}$.

Now, the marginal state

$$\begin{aligned} \rho_{\mathbf{A}} &:= \operatorname{Tr}_{\mathbf{R}}\left[|\Psi\rangle\langle\Psi|_{\mathbf{AR}}\right] \\ &= \frac{1}{G_{N,d}} \sum_i \left(|S\rangle\langle S|_{\mathbf{A}}^{\otimes N/d}\right)_i \end{aligned} \tag{59}$$

is invariant under permutations. Hence, the Schur lemma implies the relation

$$\rho_{\mathbf{A}} = \frac{Q_{\lambda_0}}{m_{\lambda_0}}\,. \tag{60}$$

Since $|\Psi\rangle_{\mathbf{AR}}$ is a purification of $\rho_{\mathbf{A}}$, we conclude that $|\Psi\rangle_{\mathbf{AR}}$ is a maximally entangled state between R and the multiplicity system M_{λ_0}. Hence, $|\Psi\rangle_{\mathbf{AR}}$ coincides with the optimal input state of Proposition *3*.

Moreover, comparing Equations (59) and (60) we obtain that the rank of $\rho_{\mathbf{A}}$ is equal to the multiplicity m_{λ_0}. Since the rank of $\rho_{\mathbf{A}}$ is the number of linearly independent vectors of the form $\left(|S\rangle_{\mathbf{A}}^{\otimes N/d}\right)_i$, we conclude that the number of such vectors is m_{λ_0}. Finally, m_{λ_0} can be expressed as in Equation (54). □

Supplementary Note 3: Optimal classical strategy for k causal hypotheses

Here we provide the optimal classical strategy for the case where exactly one out of k possible variables $B_1, B_2, \ldots, B_k$ is the causal intermediary of A. The result is stated in the following

Lemma 4. The minimum error probability in the identification of the causal intermediary among $k \geq 2$ alternatives is

$$p_{\rm err}^{\rm C} = \frac{k-1}{2d^{N-1}} + O\left(\frac{1}{d^{2N}}\right) .$$

Proof. Suppose that the i-th output variable is not the causal intermediary. The probability that it takes values compatible with a permutation is $P(d,v)/d^N$, where v is the number of distinct values of A probed in the experiment and $P(d,v) = d!/(d-v)!$ is the number of injective functions from a v-element set to a d-element set.

Hence, the probability that the i-th variable—and *only* the i-th variable—is confusable with the true causal intermediary is

$$p_i = \frac{P(d,v)}{d^N} \left[1 - \frac{P(d,v)}{d^N}\right]^{k-2} . \tag{61}$$

Similarly, the probability that that variables $i_1,\ i_2, \dots, i_t$ (and *only* variables $i_1,\ i_2, \dots, i_t$) are confusable with the true causal intermediary is

$$p_{i_1 i_2 \dots i_t} = \left[\frac{P(d,v)}{d^N}\right]^t \left[1 - \frac{P(d,v)}{d^N}\right]^{k-t-1} . \tag{62}$$

When this situation arises, one has to resort to a random guess, with probability of error $t/(t+1)$. In total, the probability of error is equal to

$$\begin{aligned} p_{\rm err}^{\rm C} &= \sum_{t=1}^{k-1} \frac{t}{t+1} \binom{k-1}{t} \left[\frac{P(d,v)}{d^N}\right]^t \left[1 - \frac{P(d,v)}{d^N}\right]^{k-t-1} \\ &= \frac{(k-1)\,P(d,v)}{2d^N} + O\left(\frac{1}{d^{2N}}\right) . \end{aligned} \tag{63}$$

Since the coefficient $P(d,v)$ is minimum when $v=1$, the optimal strategy is to initialize all input variables in the same value, thus obtaining probability of error $p_{\rm err}^{\rm C} = \frac{k-1}{2d^{N-1}} + O\left(\frac{1}{d^{2N}}\right)$. □

Supplementary Note 4: Optimal quantum strategy for k hypotheses without reference system

Here we provide the best strategy among all quantum strategies that do not use a reference system.

Lemma 5. The best quantum strategy without reference system is to divide the N input variables into N/d groups of d elements each and, within each group, to prepare the singlet state

$$|S_d\rangle = \frac{1}{\sqrt{d!}} \sum_{k_1,k_2,\cdots,k_d} \epsilon_{k_1 k_2 \dots k_d}\, |k_1\rangle|k_2\rangle \cdots |k_d\rangle \tag{64}$$

where $\epsilon_{k_1 k_2 \dots k_d}$ is the totally antisymmetric tensor and the sum ranges over all vectors in the computational basis. The corresponding error probability is

$$p_{\rm err}^{\rm QC} = \frac{k-1}{2d^N} + O\left(\frac{1}{d^{2N}}\right) . \tag{65}$$

Proof. Let us denote by x the "true causal intermediary", namely the quantum system B_x whose state depends on the state of A, and by $\mathcal{C}_{x,U}$ the channel defined by the relation

$$\mathcal{C}_{x,U}(\rho) = \big[\mathcal{U}(\rho)\big]_x \otimes \left(\frac{I}{d}\right)_{\overline{x}}^{\otimes(k-1)} , \tag{66}$$

where the subscript x indicates that the operator $\mathcal{U}(\rho)$ acts on the Hilbert space of system B_x and the subscript $\overline{x}$ indicates that the operator acts on the Hilbert space of the remaining $k-1$ systems.

By the same arguments used in Lemma *2*, the discrimination of the causal hypotheses can be reduced to the discrimination of the channels

$$\mathcal{C}_x^{(N)} = \int \mathrm{d}U \, \mathcal{C}_{x,U}^{\otimes N} \,, \qquad x \in \{1, \dots, k\} \,. \tag{67}$$

Again, one can show that, for every reference system R, the optimal state can be chosen of the form

$$\rho = \frac{P_{\lambda_0}}{d_{\lambda_0}} \otimes \Psi_{\lambda_0 R} \,, \tag{68}$$

where P_{λ_0} is the projector on the $\mathsf{SU}(d)$ representation space with Young diagram λ_0, $d_{\lambda_0} = \mathrm{Tr}[P_{\lambda_0}]$, and $\Psi_{\lambda_0 R}$ is a pure state of the composite system $\mathcal{M}_{\lambda_0} \otimes \mathcal{H}_R$, $\mathcal{M}_{\lambda_0}$ being the $\mathsf{SU(d)}$ multiplicity space associated to λ_0.

Here we consider the case where the reference system R is trivial. In this case, the problem is to distinguish among the states

$$\rho_x := \left(\frac{P_{\lambda_0}}{d_{\lambda_0}} \otimes \Psi_{\lambda_0} \right)_x \otimes \left(\frac{I}{d} \right)_{\overline{x}}^{\otimes N \, (k-1)} \qquad x \in \{1, \dots, k\} \,. \tag{69}$$

Using the Yuen-Kennedy-Lax formula [4], the maximum success probability in distinguishing among these states is

$$p_{\mathrm{succ}} = \min \left\{ \mathrm{Tr}[\Gamma] \mid \Gamma \geq \frac{1}{k} \, \rho_x \,, \quad \forall x \in \{1, \dots, k\} \right\} .$$

Note that the states $\{\rho_x \,, k = 1, \dots, k\}$ commute. Hence, they can be diagonalized in the same basis and the operator Γ can be chosen to be diagonal in that basis without loss of generality. With a similar argument, one can restrict the search for the optimal Γ over the operators of the form

$$\Gamma = \bigoplus_{\lambda_1, \lambda_2, \dots, \lambda_k} P_{\lambda_1} \otimes P_{\lambda_2} \otimes \dots \otimes P_{\lambda_k} \otimes \Gamma_{\lambda_1, \dots, \lambda_k} \,, \tag{70}$$

where $\Gamma_{\lambda_1, \dots, \lambda_k}$ is an operator acting on the tensor product space $\mathcal{M}_{\lambda_1} \otimes \mathcal{M}_{\lambda_2} \otimes \dots \otimes \mathcal{M}_{\lambda_k}$. Note that the operators $\Gamma_{\lambda_1, \dots, \lambda_k}$ can be set to zero for all k-tuples $(\lambda_1, \dots, \lambda_k)$ such that $\lambda_i \neq \lambda_0$ for every $i \in \{1, \dots, k\}$. Now, suppose that $\lambda_i = \lambda_0$ and $\lambda_j \neq 0$ for the remaining $j \neq i$. In this case, we must have

$$\Gamma_{\lambda_1, \dots, \lambda_{i-1} \lambda_0 \lambda_{i+1} \dots \lambda_k} \ \geq \frac{1}{k d_{\lambda_0} d^{N(k-1)}} \, Q_{\lambda_1} \otimes \dots \otimes Q_{\lambda_{i-1}} \otimes \Psi_{\lambda_0} \otimes Q_{\lambda_{i+1}} \otimes \dots \otimes Q_{\lambda_k} \,, \tag{71}$$

where Q_λ is the identity operator on the multiplicity space $\mathcal{M}_\lambda$. Taking the trace on both sides, we obtain the relation

$$\mathrm{Tr} \left[\Gamma_{\lambda_1, \dots, \lambda_{i-1} \lambda_0 \lambda_{i+1} \dots \lambda_k} \right] \ \geq \frac{1}{k d_{\lambda_0} d^{N(k-1)}} \, m_{\lambda_1} \dots m_{\lambda_{i-1}} \, m_{\lambda_{i+1}} \dots m_{\lambda_k} \,. \tag{72}$$

Similar bounds can be found for the operators $\Gamma_{\lambda_1, \dots, \lambda_k}$ where two or more indices are equal to λ_0. For example, consider the terms where $\lambda_i = \lambda_j = \lambda_0$, while $\lambda_l \neq 0$ for the remaining values of l. In this case, we have the conditions

$$\Gamma_{\lambda_1, \dots, \lambda_k} \geq \frac{1}{k d_{\lambda_0} d^{N(k-1)}} \left(\Psi_{\lambda_0} \otimes Q_{\lambda_0} \right)_{ij} \otimes \left(Q_\lambda \right)_{\overline{ij}} \tag{73}$$

$$\Gamma_{\lambda_1, \dots, \lambda_k} \geq \frac{1}{k d_{\lambda_0} d^{N(k-1)}} \left(Q_{\lambda_0} \otimes \Psi_{\lambda_0} \right)_{ij} \otimes \left(Q_\lambda \right)_{\overline{ij}} , \tag{74}$$

where we introduced the shorthand notation

$$\left(Q_\lambda \right)_{\overline{ij}} := Q_{\lambda_1} \otimes \dots \otimes Q_{\lambda_{i-1}} \otimes Q_{\lambda_{i+1}} \otimes \dots \otimes Q_{\lambda_{j-1}} \otimes Q_{\lambda_{j+1}} \otimes \dots \otimes Q_{\lambda_k} \,. \tag{75}$$

We now combine conditions (73) and (74) can be combined into a single condition. To this purpose, we expand Q_{λ_0} as

$$Q_{\lambda_0} = \Psi_{\lambda_0} + \Psi_{\lambda_0}^{\perp} \,,$$

which allows for rewriting (73) and (74) as

$$\Gamma_{\lambda_1,\ldots,\lambda_k} \geq \frac{1}{kd_{\lambda_0} d^{N(k-1)}} \left(\Psi_{\lambda_0} \otimes \Psi_{\lambda_0} + \Psi_{\lambda_0} \otimes \Psi^{\perp}_{\lambda_0}\right)_{ij} \otimes \left(Q_\lambda\right)_{\overline{ij}} \tag{76}$$

$$\Gamma_{\lambda_1,\ldots,\lambda_k} \geq \frac{1}{kd_{\lambda_0} d^{N(k-1)}} \left(\Psi_{\lambda_0} \otimes \Psi_{\lambda_0} + \Psi^{\perp}_{\lambda_0} \otimes \Psi_{\lambda_0}\right)_{ij} \otimes \left(Q_\lambda\right)_{\overline{ij}} . \tag{77}$$

Now, since $\Psi_{\lambda_0} \otimes \Psi^{\perp}_{\lambda_0}$ and $\Psi^{\perp}_{\lambda_0} \otimes \Psi_{\lambda_0}$ are orthogonal vectors, it is also true that

$$\Gamma_{\lambda_1,\ldots,\lambda_k} \geq \frac{1}{kd_{\lambda_0} d^{N(k-1)}} \left(\Psi_{\lambda_0} \otimes \Psi_{\lambda_0} + \Psi_{\lambda_0} \otimes \Psi^{\perp}_{\lambda_0} + \Psi^{\perp}_{\lambda_0} \otimes \Psi_{\lambda_0}\right)_{ij} \otimes \left(Q_\lambda\right)_{\overline{ij}},$$

which can be rewritten as

$$\Gamma_{\lambda_1,\ldots,\lambda_k} \geq \frac{1}{kd_{\lambda_0} d^{N(k-1)}} \left(Q_{\lambda_0} \otimes Q_{\lambda_0} - \Psi^{\perp}_{\lambda_0} \otimes \Psi^{\perp}_{\lambda_0}\right)_{ij} \otimes \left(Q_\lambda\right)_{\overline{ij}} . \tag{78}$$

Tracing on both sides, one obtains

$$\mathrm{Tr}\left[\Gamma_{\lambda_1,\ldots,\lambda_k}\right] \geq \frac{1}{kd_{\lambda_0} d^{N(k-1)}} \left(2m_{\lambda_0} - 1\right) \left(\prod_{l\neq i,j} m_{\lambda_l}\right) . \tag{79}$$

Likewise, a term with $\lambda_{i_1} = \lambda_{i_2} = \cdots = \lambda_{i_t} = \lambda_0$ and all the remaining λ_l different from λ_0 will satisfy the condition

$$\Gamma_{\lambda_1,\ldots,\lambda_k} \geq \frac{1}{kd_{\lambda_0} d^{N(k-1)}} \left(Q^{\otimes t}_{\lambda_0} - \Psi^{\perp\otimes t}_{\lambda_0}\right)_{i_1\ldots i_t} \otimes \left(Q_\lambda\right)_{\overline{i_1\ldots i_t}}, \tag{80}$$

leading to the inequality

$$\mathrm{Tr}\left[\Gamma_{\lambda_1,\ldots,\lambda_k}\right] \geq \frac{1}{kd_{\lambda_0} d^{N(k-1)}} \left[m^t_{\lambda_0} - (m_{\lambda_0} - 1)^t\right] \prod_{l\neq i_1,\ldots,i_t} m_{\lambda_l} . \tag{81}$$

Note that one can choose the operator Γ in such a way that the equality holds in all bounds. With this choice, the probability of success is

$$\begin{aligned} p_{\text{succ}} &= \sum_{\lambda_1,\ldots,\lambda_k} d_{\lambda_1} \ldots d_{\lambda_k} \ \mathrm{Tr}[\Gamma_{\lambda_1,\ldots\,\lambda_k}] \\ &= \sum_{t=1}^{k} \binom{k}{t} \frac{(d_{\lambda_0} m_{\lambda_0})^t}{kd_{\lambda_0} d^{N(k-1)}} \left[1 - \left(1 - \frac{1}{m_{\lambda_0}}\right)^t\right] \left(d^N - d_{\lambda_0} m_{\lambda_0}\right)^{k-t} \\ &= \frac{d^N}{kd_{\lambda_0}} \sum_{t=1}^{k} \binom{k}{t} p^t_{\lambda_0} \left(1 - p_{\lambda_0}\right)^{k-t} \left[1 - \left(1 - \frac{1}{m_{\lambda_0}}\right)^t\right] , \end{aligned} \tag{82}$$

having defined the Schur-Weyl measure $p_\lambda := d_\lambda m_\lambda / d^N$.

Expanding the term in square brackets, we obtain

$$\begin{aligned} p_{\text{succ}} &= \frac{d^N}{kd_{\lambda_0}} \sum_{t=1}^{k} \binom{k}{t} p^t_{\lambda_0} \left(1 - p_{\lambda_0}\right)^{k-t} \left[\sum_{s=1}^{t} \binom{t}{s} \frac{(-1)^{s+1}}{m^s_{\lambda_0}}\right] \\ &= \frac{d^N}{kd_{\lambda_0}} \sum_{s=1}^{k} \frac{(-1)^{s+1}}{m^s_{\lambda_0}} \left[\sum_{t=s}^{k} \binom{k}{t} \binom{t}{s} p^t_{\lambda_0} \left(1 - p_{\lambda_0}\right)^{k-t}\right] \\ &= \frac{d^N}{kd_{\lambda_0}} \sum_{s=1}^{k} \frac{(-1)^{s+1} p^s_{\lambda_0}}{m^s_{\lambda_0}} \binom{k}{s} \\ &= \frac{d^N}{kd_{\lambda_0}} \left[1 - \left(1 - \frac{p_{\lambda_0}}{m_{\lambda_0}}\right)^k\right] \\ &= 1 - \frac{(k-1) d_{\lambda_0}}{2d^N} + O\left[\left(\frac{d_{\lambda_0}}{d^N}\right)^2\right] . \end{aligned} \tag{83}$$

Hence, the error probability is

$$p_{\rm err} = \frac{(k-1)d_{\lambda_0}}{2d^N} + O\left[\left(\frac{d_{\lambda_0}}{d^N}\right)^2\right] . \tag{84}$$

Again, the optimal choice for N multiple of d is to pick λ_0 to be the trivial representation of $\mathsf{SU}(d)$, in which case the error probability is

$$p_{\rm err} = \frac{(k-1)}{2d^N} + O\left(\frac{1}{d^{2N}}\right) . \tag{85}$$

Note that, however, the choice of representation λ_0 does not affect the asymptotic rate: indeed, for every λ_0 we have

$$\begin{aligned} R &= -\liminf_{N\to\infty} \frac{\log p_{\rm err}}{N} \\ &= \log d - \liminf_{N\to\infty} \frac{\log[(k-1)\, d_{\lambda_0}/2]}{N} \\ &= \log d \\ &\equiv R_{\rm C} . \end{aligned} \tag{86}$$

Note also that the rate is independent of the number of hypotheses, as in the case of the Chernoff bound for quantum states [8]. □

Supplementary Note 5: Optimal quantum strategy for k causal hypotheses with arbitrary reference system

Here we provide the optimal quantum strategy using a reference system. We will prove the following lemma:

Lemma 6. The optimal input state is

$$|\rho\rangle = \frac{1}{\sqrt{G_{N,d}}} \sum_{i=1}^{G_{N,d}} \left(|S_d\rangle^{\otimes N/d}\right)_i \otimes |i\rangle , \tag{87}$$

where i labels the different ways to divide N identical objects into groups of d elements, $G_{N,d} = \frac{N!}{(d!)^{N/d}(N/d)!}$ is the total number of such ways, $\left(|S_d\rangle^{\otimes N/d}\right)_i$ is the product of N/d singlet states arranged according to the configuration i, and $\{|i\rangle\,,\, i=1,\dots,G_{N,d}\}$ are orthogonal states of the reference system, chosen to be of dimension equal to or larger than $G_{N,d}$. The corresponding error probability is upper bounded as

$$p^{\rm Q}_{\rm err}(r) \le \frac{k-1}{2d^N m(N,d)} \tag{88}$$

where $m(N,d)$ is the dimension of the multiplicity space of the trivial representation, given by (for N/d being an integer)

$$m(N,d) = d^N \left[\frac{d^{\frac{d^2}{2}}\, e^{\frac{d(d-1)}{2}} \prod_{i=1}^d (d-i)!}{(2\pi)^{\frac{d-1}{2}}\, N^{\frac{d^2-1}{2}}}\right] c(N) , \tag{89}$$

with $\lim_{N\to\infty} c(N) = 1$.

The proof consists of four steps:

Step 1: reduction to the permutation register. We apply N uses of the channel $\mathcal{C}_x$ to a state of the optimal form (31), where the pure state $|\Psi_{\lambda_0}\rangle$ is set to be the maximally entangled state $|\Phi_{\lambda_0}\rangle = \sum_{i=1}^{m_{\lambda_0}} |i\rangle \otimes |i\rangle/\sqrt{m_{\lambda_0}}$. The output state is

$$\rho_x^{\rm out} = \left(\frac{P_{\lambda_0}}{d_{\lambda_0}} \otimes \Phi_{\lambda_0}\right)_x \otimes \left(\frac{I}{d}\right)_{\overline{x}}^{\otimes N(k-1)} , \tag{90}$$

where the subscript x indicates that the corresponding operator acts on the N Hilbert spaces with label x (and on the reference), while the subscript $\overline{x}$ indicates that the corresponding operator acts on all systems except those with label x.

Breaking down the identity operator as $I = (P_{\lambda_0} \otimes Q_{\lambda_0}) \oplus (I - P_{\lambda_0} \otimes Q_{\lambda_0})$, we can decompose $\rho_x^{\rm out}$ into orthogonal blocks where exactly l output systems are in the sector λ_0. Explicitly, we have

$$\rho_x^{\rm out} = \bigoplus_{l=1}^{k} \bigoplus_{\mathsf{A} \in \mathsf{S}_l} q(\mathsf{A}|x) \left(\rho_{\mathsf{A},x} \otimes \chi_{\overline{\mathsf{A}}} \right) , \tag{91}$$

where S_l denotes the set of all l-element subsets of $\{1, 2, \dots, k\}$, $\rho_{x,\mathsf{A}}$ is the quantum state defined by

$$\rho_{\mathsf{A},x} = \left(\frac{P_{\lambda_0}}{d_{\lambda_0}} \otimes \Phi_{\lambda_0} \right)_x \otimes \left[\bigotimes_{i \in \mathsf{A}\, , i \neq x} \left(\frac{P_{\lambda_0}}{d_{\lambda_0}} \otimes \frac{Q_{\lambda_0}}{m_{\lambda_0}} \right)_i \right] , \tag{92}$$

$\chi_{\overline{\mathsf{A}}}$ is the quantum state defined by

$$\chi_{\overline{\mathsf{A}}} = \bigotimes_{i \notin \mathsf{A}} \left(\frac{I^{\otimes N} - P_{\lambda_0} \otimes Q_{\lambda_0}}{d^N - d_{\lambda_0} m_{\lambda_0}} \right)_i , \tag{93}$$

and $q(\mathsf{A}|x)$ is the conditional probability distribution defined by

$$q(\mathsf{A}|x) = \begin{cases} p_{\lambda_0}^{l-1} \, (1 - p_{\lambda_0})^{k-l} & \text{for } x \in \mathsf{A} \\ 0 & \text{for } x \notin \mathsf{A} \, , \end{cases} \tag{94}$$

$p_\lambda := d_\lambda m_\lambda / d^N$ being the Schur-Weyl measure,

From Eq. (91) one can see that blocks with different values of l and/or different subsets A are orthogonal for every value of x. Hence, one can extract first the information about the block and then the information about x. Mathematically, this means performing a non-demolition measurement with outcomes (l, A), which projects the state into the block labelled by (l, A). When such a measurement is performed on the state $\rho_x^{\rm out}$, the outcome (l, A) can occur only if A contains x—in which case the probability of occurrence is $q(\mathsf{A}|x)$. Conditionally on the outcome, the system is left in the state $\rho_{\mathsf{A},x} \otimes \chi_{\overline{\mathsf{A}}}$ and the problem is to identify x within the set A. Hence, the probability of success for fixed x is

$$p_{\rm succ}(x) = \sum_{l=1}^{k} \sum_{\mathsf{A} \in \mathsf{S}_l} q(\mathsf{A}|x) \; p_{\rm succ}^{(\mathsf{A})}(x) \, , \tag{95}$$

where $p_{\rm succ}^{(\mathsf{A})}(x)$ is the probability of correctly identifying the state $\rho_{\mathsf{A},x} \otimes \chi_{\overline{\mathsf{A}}}$.

Note that, for $x \in \mathsf{A}$, the optimal success probability $p_{\rm succ}^{(\mathsf{A})}(x)$ does not depend on the specific subset A, but only on its cardinality l: indeed, $p_{\rm succ}^{(\mathsf{A})}(x)$ coincides with the probability $p_{\rm succ}^{(l)}(x)$ of correctly identifying the label of the states

$$\sigma_x = \Phi_x \otimes \left(\frac{I_m}{m} \right)_{\overline{x}}^{\otimes l-1} , \qquad x \in \{1, 2, \dots, l\} \, , \tag{96}$$

where we used the shorthand notation $\Phi_x := (\Phi_{\lambda_0})_x$, and used I_m to denote the identity matrix in dimension m, with $m = m_{\lambda_0}$ (these are the states that arise from Eq. (92) after discarding the representation spaces). We denote by $p_{\rm succ}^{(l)}$ the average success probability

$$p_{\rm succ}^{(l)} = \frac{1}{l} \sum_{x=1}^{l} p_{\rm succ}^{(l)}(x) \, . \tag{97}$$

Averaging the success probability (95) over x, we obtain

$$
\begin{aligned}
p_{\rm succ} &= \frac{1}{k}\sum_{x=1}^{k} p_{\rm succ}(x) \\
&= \frac{1}{k}\sum_{x=1}^{k}\sum_{l=1}^{k}\sum_{\mathsf{A}\in\mathsf{S}_l} q(\mathsf{A}|x)\; p^{(\mathsf{A})}_{\rm succ}(x) \\
&= \frac{1}{k}\sum_{l=1}^{k}\sum_{\mathsf{A}\in\mathsf{S}_l}\sum_{x\in\mathsf{A}} p_{\lambda_0}^{l-1}\,(1-p_{\lambda_0})^{k-l}\; p^{(\mathsf{A})}_{\rm succ}(x) \\
&= \frac{1}{k}\sum_{l=1}^{k}\sum_{\mathsf{A}\in\mathsf{S}_l} p_{\lambda_0}^{l-1}\,(1-p_{\lambda_0})^{k-l}\, l\, p^{(l)}_{\rm succ} \\
&= \frac{1}{k}\sum_{l=1}^{k}\left|\mathsf{S}_l\right|\, p_{\lambda_0}^{l-1}\,(1-p_{\lambda_0})^{k-l}\, l\, p^{(l)}_{\rm succ} \\
&= \frac{1}{k}\sum_{l=1}^{k}\binom{k}{l}\, p_{\lambda_0}^{l-1}\,(1-p_{\lambda_0})^{k-l}\, l\, p^{(l)}_{\rm succ}\,.
\end{aligned}
\tag{98}
$$

The next step is to compute $p^{(l)}_{\rm succ}$.

Step 2: reduction to type states. The state σ_x in Eq. (96) is the product of a maximally entangled state and a $(l-1)$ copies of the maximally mixed state. The latter can be diagonalized as

$$
\left(\frac{I_m}{m}\right)_{\overline{x}}^{\otimes(l-1)} = \frac{1}{m^{l-1}}\sum_{\mathbf{j}}|\mathbf{j}\rangle\langle\mathbf{j}|\,, \tag{99}
$$

where $|\mathbf{j}\rangle$ is the basis vector $|\mathbf{j}\rangle = |j_1\rangle\otimes|j_2\rangle\otimes\cdots\otimes|j_{l-1}\rangle$ corresponding to the sequence $\mathbf{j}=(j_1,j_2,\dots,j_{l-1})\in\{1,\dots,m\}^{\times(l-1)}$.

Now, let us introduce the shorthand

$$
|\Phi_{x,\mathbf{j}\rangle} := |\Phi\rangle_x\otimes|\mathbf{j}\rangle_{\overline{x}}\,. \tag{100}
$$

Note that for $x\le y$ one has

$$
\langle\Phi_{x,\mathbf{j}}|\Phi_{y,\mathbf{k}}\rangle = \begin{cases}
1 & x=y\,, \quad \mathbf{j}=\mathbf{k} \\[1ex]
\frac{1}{m} & x\neq y\,, \quad \begin{array}{l} j_i=k_i\,,\ \forall i<x \\ j_i=k_{i+1}\,,\forall x\le i<y-1 \\ j_{y-1}=k_x \\ j_i=k_i\,,\forall i\ge y \end{array} \\[1ex]
0 & \text{otherwise}\,.
\end{cases} \tag{101}
$$

Let $\mathbf{n}=(n_1,n_2,\dots,n_m)$ be a partition of $l-1$ into m nonnegative integers. Recall that the sequence $\mathbf{j}=(j_1,j_2,\dots,j_{l-1})$ is said to be *of type* $\mathbf{n}$ if it n_1 entries of $\mathbf{j}$ are equal to 1, n_2 entries are equal to 2, and so on. Eq. (101) tells us that the vectors $|\Phi_{x,\mathbf{j}}\rangle$ and $|\Phi_{y,\mathbf{k}}\rangle$ are orthogonal whenever the sequences $\mathbf{j}$ and $\mathbf{k}$ are of different type. Using this fact, we can define the orthogonal subspaces

$$
\mathcal{H}_{\mathbf{n}} = \mathsf{Span}\Big\{|\Phi_{x,\mathbf{j}}\rangle \mid x\in\{1,\dots,l\}\,,\ \mathbf{j}\in\mathsf{S}_{\mathbf{n}}\Big\}\,, \tag{102}
$$

where $\mathsf{S}_{\mathbf{n}}$ is the set of all sequences of length $l-1$ and of type $\mathbf{n}$. Hence, we can decompose the states σ_x in Eq. (96) as

$$
\sigma_x = \bigoplus_{\mathbf{n}} p(\mathbf{n})\,\sigma_{\mathbf{n},x}\,, \tag{103}
$$

with

$$p(\mathbf{n}) = \frac{C_{\mathbf{n}}}{m^{l-1}} \qquad \text{and} \qquad \sigma_{\mathbf{n},x} = \frac{1}{C_{\mathbf{n}}} \sum_{\mathbf{j}\in \mathsf{S}_{\mathbf{n}}} |\Phi_{x,\mathbf{j}}\rangle\langle\Phi_{x,\mathbf{j}}| \,, \tag{104}$$

where $C_{\mathbf{n}} = (l-1)!/[n_1!n_2!\cdots n_m!]$ is the number of sequences of type $\mathbf{n}$.

Eq. (103) tells us that, in order to distinguish the states σ_x, one can perform an orthogonal measurement that projects on the subspaces $\{\mathcal{H}_{\mathbf{n}}\}$ (102). If the measurement outcome is $\mathbf{n}$, one is left with the task of distinguishing among the states $\sigma_{\mathbf{n},x}$. The success probability of this strategy is

$$p_{\rm succ}^{(l)} = \sum_{\mathbf{n}} p(\mathbf{n})\; p_{\rm succ}^{(\mathbf{n})} \,, \tag{105}$$

where $p_{\rm succ}^{(\mathbf{n})}$ is the probability of correctly distinguishing the states $\{\sigma_{\mathbf{n},x} \mid x \in \{1,\dots,l\}\}$.

Step 3: lower bound on the probability of success. The probability of correctly distinguishing the states $\{\sigma_{\mathbf{n},x} \mid x \in \{1,\dots,l\}\}$ is lower bounded by the probability of correctly distinguishing among all their eigenstates

$$\Big\{|\Phi_{x,\mathbf{j}}\rangle \mid x\in\{1,\dots,l\}\,,\ \mathbf{j}\in\mathsf{S}_{\mathbf{n}}\Big\}\,. \tag{106}$$

Note that the total number of states is $l\,C_{\mathbf{n}}$.

We now construct a measurement that distinguishes these states with high success probability. The measurement is constructed through a Grahm-Schmidt orthogonalization procedure. We define a first batch of $C_{\mathbf{n}}$ vectors as

$$|\Psi_{1,\mathbf{j}}\rangle := |\Phi_{1,\mathbf{j}}\rangle \qquad \mathbf{j}\in\mathsf{S}_{\mathbf{n}}\,. \tag{107}$$

This definition is well-posed, because the above vectors are orthonormal, due to Eq. (101).

A second batch of vectors is constructed from the vectors $\{|\Phi_{2,\mathbf{j}}\rangle\,, \mathbf{j}\in\mathsf{S}_{\mathbf{n}}\}$ via the Grahm-Schmidt procedure, which yields

$$|\Psi_{2,\mathbf{j}}\rangle := \frac{|\Phi_{2,\mathbf{j}}\rangle - \frac{1}{m}\,|\Phi_{1,\mathbf{j}^{12}}\rangle}{\sqrt{1-\frac{1}{m^2}}}\,, \tag{108}$$

where $\mathbf{j}^{12}$ is the sequence such that $\langle\Phi_{1,\mathbf{j}^{12}}|\Phi_{2,\mathbf{j}}\rangle = 1/m$.

A third batch of vectors is constructed from the vectors $\{|\Phi_{2,\mathbf{j}}\rangle\,, \mathbf{j}\in\mathsf{S}_{\mathbf{n}}\}$. Now, the Grahm-Schmidt procedure yields

$$|\Psi_{3,\mathbf{j}}\rangle := \frac{|\Phi_{3,\mathbf{j}}\rangle - \frac{1}{m}\,|\Phi_{2,\mathbf{j}^{23}}\rangle - \frac{1}{m}\,|\Phi_{1,\mathbf{j}^{13}}\rangle}{\sqrt{1-\frac{2}{m^2}}} + O\left(\frac{1}{m^2}\right)\,|\Gamma_{3,\mathbf{j}}\rangle + O\left(\frac{1}{m^3}\right)\,|{\rm Rest}_{3,\mathbf{j}}\rangle\,, \tag{109}$$

where $|\Gamma_{3,\mathbf{j}}\rangle$ is a vector of the form $|\Phi_{1,\mathbf{k}}\rangle$ for some suitable $\mathbf{k}$ and $|{\rm Rest}_{3,\mathbf{j}}\rangle$ is a suitable unit vector, which is irrelevant for computing the leading order of the success probability.

In general, the x-th batch of vectors is

$$|\Psi_{x,\mathbf{j}}\rangle := \frac{|\Phi_{x,\mathbf{j}}\rangle - \frac{1}{m}\,\sum_{y=1}^{x-1}|\Phi_{y,\mathbf{j}^{yx}}\rangle}{\sqrt{1-\frac{x-1}{m^2}}} + O\left(\frac{1}{m^2}\right)\,|\Gamma_{x,\mathbf{j}}\rangle + O\left(\frac{1}{m^3}\right)\,|{\rm Rest}_{x,\mathbf{j}}\rangle\,, \tag{110}$$

where $|\Gamma_{x,\mathbf{j}}\rangle$ is a normalized combination of vectors of the form $|\Phi_{z,\mathbf{k}_z}\rangle$, $z < x-2$, while $|{\rm Rest}_{x,\mathbf{j}}\rangle$ is a suitable unit vector.

Note that one has

$$\langle\Phi_{x,\mathbf{j}}|\Psi_{x,\mathbf{j}}\rangle = \sqrt{1-\frac{x-1}{m^2}} + O\left(\frac{1}{m^3}\right)\,, \qquad \forall x\in\{1,\dots,l\}\,, \quad \forall \mathbf{j}\in\mathsf{S}_{\mathbf{n}}\,, \tag{111}$$

having used the fact that the product $\langle\Phi_{x,{\rm j}}|\Gamma_{x,\mathbf{j}}\rangle$ is $O(1/m)$.

Using Eq. (111), we can now evaluate the probability of correctly distinguishing the states $\{|\Phi_{x,\mathbf{j}}\rangle\}$. On average over all possible states, the probability of success is

$$\begin{aligned}p_{\text{succ}}^{(\mathbf{n})} &= \frac{1}{lC_{\mathbf{n}}}\sum_{x=1}^{l}\sum_{\mathbf{j}\in\mathsf{S}_{\mathbf{n}}}\left|\langle\Psi_{x,\mathbf{j}}|\Phi_{x,\mathbf{j}}\rangle\right|^2\\ &= \frac{1}{lC_{\mathbf{n}}}\sum_{x=1}^{l}\sum_{\mathbf{j}\in\mathsf{S}_{\mathbf{n}}}\left[1-\frac{x-1}{m^2}+O\left(\frac{1}{m^3}\right)\right]\\ &= \frac{1}{l}\sum_{x=1}^{l}\left[1-\frac{x-1}{m^2}+O\left(\frac{1}{m^3}\right)\right]\\ &= 1-\frac{l-1}{2m^2}+O\left(\frac{1}{m^3}\right). \end{aligned} \tag{112}$$

Since measuring on the basis $\{|\Psi_{x,\mathbf{j}}\rangle\}$ is not necessarily the optimal strategy, we arrived at the lower bound

$$p_{\text{succ}}^{(\mathbf{n})} \ge 1-\frac{l-1}{2m^2}+O\left(\frac{1}{m^3}\right). \tag{113}$$

Note that the (leading order of the) r.h.s. is independent of the type $\mathbf{n}$.

Step 4: putting everything together. Combining the results obtained so far, we can lower bound the success probability in distinguishing among k causal structures. Inserting the lower bound (113) into Eq. (105), we obtain

$$\begin{aligned}p_{\text{succ}}^{(l)} &= \sum_{\mathbf{n}} p(\mathbf{n})\, p_{\text{succ}}^{\mathbf{n}}\\ &\ge 1-\frac{l-1}{2m^2}+O\left(\frac{1}{m^3}\right).\end{aligned}$$

Then, we can insert the above bound into Eq. (98). Reverting to the full notation $m_{\lambda_0}\equiv m$, we obtain

$$\begin{aligned}p_{\text{succ}} &= \frac{1}{k}\sum_{l=1}^{k}\binom{k}{l}\, p_{\lambda_0}^{l-1}\,(1-p_{\lambda_0})^{k-l}\, l\, p_{\text{succ}}^{(l)}\\ &\ge \frac{1}{k}\sum_{l=1}^{k}\binom{k}{l}\, l\, p_{\lambda_0}^{l-1}\,(1-p_{\lambda_0})^{k-l}\left[1-\frac{l-1}{2m_{\lambda_0}^2}+O\left(\frac{1}{m_{\lambda_0}^3}\right)\right]\\ &= 1-\frac{(k-1)p_{\lambda_0}}{2m_{\lambda_0}^2}+O\left(\frac{1}{m_{\lambda_0}^3}\right)\\ &= 1-\frac{k-1}{2d^N}\frac{d_{\lambda_0}}{m_{\lambda_0}}+O\left(\frac{1}{m_{\lambda_0}^3}\right). \end{aligned} \tag{114}$$

Hence, the error probability of the optimal quantum strategy is upper bounded as

$$p_{\text{err}} \le \frac{k-1}{2d^N}\frac{d_{\lambda_0}}{m_{\lambda_0}}+O\left(\frac{1}{m_{\lambda_0}^3}\right). \tag{115}$$

Recalling that the ratio d_λ/m_λ is minimised by the representation with "minimal" Young diagram (in the majorisation order), we conclude that, when N is a multiple of d, the optimal error probability satisfies the bound

$$p_{\text{err}} \le \frac{k-1}{2d^N\, m(N,d)}+O\left(\frac{1}{m_{\lambda_0}^3}\right), \qquad \text{with} \qquad m(N,d)=d^N\left[\frac{d^{\frac{d^2}{2}}\, e^{\frac{d(d-1)}{2}}\prod_{i=1}^{d}(d-i)!}{(2\pi)^{\frac{d-1}{2}}\, N^{\frac{d^2-1}{2}}}\right]c(N) \quad \text{and} \quad c(N)\to 1\,. \tag{116}$$

Hence, the asymptotic decay rate is lower bounded as

$$\begin{aligned}R_{\mathrm{Q}} &= -\lim_{N\to\infty}\frac{\log p_{\text{err}}}{N}\\ &\ge 2\log d\,.\end{aligned} \tag{117}$$

On the other hand, the r.h.s. is equal to the decay rate for $k=2$, which is a lower bound for the decay rate for $k \geq 2$. In conclusion, we obtained that the optimal decay rate is *equal* to $R_{\mathrm{Q}} = 2\log d$. $\square$

Supplementary Note 6: Quantum speedup in the identification of a cause

We consider the scenario where k quantum variables $A_1, \ldots, A_k$ are candidate causes of a given effect B. For simplicity, we assume that all variables are quantum systems of dimension $d < \infty$. The causal relation is described by a quantum channel $\mathcal{C}_{x,\mathcal{U}}$ of the form $\mathcal{C}_{x,\mathcal{U}}(\rho) = \mathcal{U}(\mathrm{Tr}_{\overline{x}}[\rho])$, where $\mathrm{Tr}_{\overline{x}}$ denotes the partial trace over all input systems except A_x, with $x \in \{1, \ldots, k\}$, and $\mathcal{U}$ is a generic unitary channel, acting on the remaining system A_x. The problem is to identify the value of x.

8. *Fixed unitary gates*

Suppose first that the unitary gate $\mathcal{U}$ is fixed. Without loss of generality, we can assume $\mathcal{U} = \mathcal{I}$, so that the channel $\mathcal{C}_{x,\mathcal{I}}$ is simply the partial trace over all systems except x. The distinguishability of the channels $\{\mathcal{C}_{x,\mathcal{I}}\}_{x=1}^k$ has been studied extensively in the optimization of port-based teleportation [9]. A simple strategy is to entangle each input system with a reference system, obtaining the output state $\rho_x := \Phi^+_{BR_x} \otimes \left(\frac{I}{d}\right)^{\otimes k-1}_{\overline{x}}$, where Φ^+ is the maximally entangled state, R_x is the x-th reference system, and the subscript $\overline{x}$ indicates that the operator $(I/d)^{\otimes(k-1)}$ acts on the Hilbert space of all reference systems except R_x.

For $k \geq d$, the optimal probability of success in distinguishing between the states $\{\rho_x\}_{x=1}^k$ is $p_{\mathrm{succ}} = d^2/(k-1+d^2)$ [9]. If the unknown process is probed for N times, the output state is $\rho_x^{\otimes N}$ and the probability of success is $p_{\mathrm{succ}} = d^{2N}/(k-1+d^{2N})$.

9. *Unknown unitary gates*

Let us consider the scenario where the unitary gate $\mathcal{U}$ is completely unknown. By the same argument as in Supplementary Note 1, the minimum worst-case error probability is equal to the minimum error probability in distinguishing between the average channels

$$\mathcal{C}_x^{(N)} = \int \mathrm{d}\mathcal{U}_1 \mathrm{d}\mathcal{U}_2 \cdots \mathrm{d}\mathcal{U}_k \quad \mathcal{C}_{x,\mathcal{I}}^{\otimes N} \circ \left(\mathcal{U}_1 \otimes \mathcal{U}_2 \otimes \cdots \otimes \mathcal{U}_k\right)^{\otimes N} . \tag{118}$$

The symmetry of the problem implies that the optimal input states are of the form

$$\rho_{\mathbf{AR}} = \frac{P_{\lambda_1}}{d_{\lambda_1}} \otimes \frac{P_{\lambda_2}}{d_{\lambda_2}} \otimes \cdots \otimes \frac{P_{\lambda_k}}{d_{\lambda_k}} \otimes \Psi_{M_{\lambda_1} M_{\lambda_2} \cdots M_{\lambda_k} R} , \tag{119}$$

where P_{λ_i} is the projector on the representation space $\mathcal{R}_{\lambda_i}$ in the tensor product $(\mathcal{H}^{\otimes N})_i$ of the N systems corresponding to variable A_i, and the subscript M_{λ_i} denotes the multiplicity space in $(\mathcal{H}^{\otimes N})_i$.

When the input variables are initialized in the state $\rho_{\mathbf{AR}}$, the output is

$$\rho_{\mathbf{BR},x} = \frac{P_{\lambda_x}}{d_{\lambda_x}} \otimes \mathrm{Tr}_{\overline{M_{\lambda_x}}} \left[\Psi_{M_{\lambda_1} M_{\lambda_2} \cdots M_{\lambda_k} R}\right] , \tag{120}$$

where $\mathrm{Tr}_{\overline{M_{\lambda_x}}}$ is the trace over all multiplicity spaces except M_{λ_x}.

We now show that the true cause can be perfectly identified using at $O(\log_d k)$ queries to the unknown process. We first provide an *exact* strategy using $\log_d k$ queries (at the leading order), and then show that the number of queries can be reduced to $1/2 \log_d k$ (at the leading order) if a small error, vanishing in the large k limit, is tolerated.

Our exact strategy disregards the reference system R. In this strategy, we prepare the multiplicity systems in the product state

$$|\Psi\rangle_{M_{\lambda_1} M_{\lambda_2} \cdots M_{\lambda_k}} = |\psi_1\rangle_{M_{\lambda_1}} \otimes |\psi_2\rangle_{M_{\lambda_2}} \otimes \cdots \otimes |\psi_k\rangle_{M_{\lambda_k}} . \tag{121}$$

We divide the indices i into L groups, labelled as $G_1, G_2, \ldots, G_L$ and assign a distinct Young diagram to each group, so that $\lambda_i = \lambda_j$ for i,j in the same group. Within each group, we choose the states $|\psi_i\rangle_{M_{\lambda_i}}$ to be orthogonal. This

choice constrains the number of indices in group G_l to be at most the dimension of the multiplicity space $\mathcal{M}_{\lambda_{G_l}}$, where λ_{G_l} is the Young diagram assigned to the group G_l. In turn, this implies that the condition

$$k \le \sum_{l=1}^{L} m_{\lambda_{G_l}} \le \sum_{\lambda} m_\lambda \tag{122}$$

must be satisfied. Both bounds can be saturated, as one can choose L to be the number of Young diagrams in the decomposition of the tensor representation $U^{\otimes N}$. On the other hand, the multiplicities are lower bounded as $m_\lambda \ge \binom{N}{\lambda}/(N+1)^{d(d-1)/2}$ where $\binom{N}{\lambda} = N!/(\lambda_1!\lambda_2!\cdots\lambda_k!)$ is the multinomial coefficient [10, 11]. Hence, we have the bound $\sum_\lambda m_\lambda \ge d^N/(N+1)^{d(d-1)/2}$, meaning that condition (122) can be satisfied with $N \ge \log_d k + O(\log\log k)$. Hence, the unknown cause can be identified with zero error using approximately $\log_d k$ queries.

We now construct a strategy that identifies the correct cause with $1/2\log_d k + O(\log\log k)$ queries and with vanishing error probability. In this strategy, all the input variables are initialized in the same sector, namely $\lambda_1 = \lambda_2 = \cdots = \lambda_k \equiv \lambda$. Specifically, we take N to be a multiple of d and choose λ to be the Young diagram corresponding to the trivial representation of $\mathsf{SU}(d)$. The strategy uses the reference system $R = M_\lambda^{\otimes k}$ and the input state

$$\rho_{\mathbf{AR}} = \left(\frac{P_\lambda}{d_\lambda}\right)^{\otimes k} \otimes \left(\Phi_\lambda^+\right)^{\otimes k}, \tag{123}$$

where Φ_λ^+ is the projector on the maximally entangled state of two identical copies of M_λ. Then, the output state is

$$\rho_{\mathbf{BR},x} = \frac{P_\lambda}{d_\lambda} \otimes \left(\Phi_\lambda^+\right)_x \otimes \left(\frac{Q_\lambda}{m_\lambda}\right)_{\overline{x}}^{\otimes(k-1)}, \tag{124}$$

where the maximally entangled state $\left(\Phi_\lambda^+\right)_x$ involves the output system B and the x-th reference system, while all the remaining reference systems are in the maximally mixed state Q_λ/m_λ. Distinguishing among the states $\rho_{\mathbf{BR},x}$ is equivalent to distinguishing the states $\left(\Phi_\lambda^+\right)_x \otimes \left(\frac{Q_\lambda}{m_\lambda}\right)_{\overline{x}}^{\otimes(k-1)}$. This problem has been solved in the context of port-based teleportation, and the minimum error probability is known to be $p_{\rm err} = (k-1)/(m_\lambda^2 + k - 1)$ [9]. Using Equation (89), we then obtain

$$p_{\rm err} \le \frac{k-1}{m_\lambda^2} = \frac{k-1}{d^{2N}}\left[\frac{(2\pi)^{d-1}\, N^{d^2-1}}{d^{d^2}\, e^{d(d-1)}\, \prod_{i=1}^{d}(d-i)!\, c(N)}\right], \tag{125}$$

with $\lim_{N\to\infty} c(N) = 1$. Hence, a vanishing error probability can be obtained by setting $N = \lceil(\log_d k)(1+\epsilon)/2\rceil$ with $\epsilon > 0$.

Supplementary Note 7: A quantum advantage in the presence of noise

Here we consider the task of identifying causal intermediaries when the cause-effect relation is obfuscated by depolarizing noise, corresponding to the channel $\mathcal{D}_p = (1-p)\mathcal{I} + pI/d$, where p is the probability of depolarization.

For simplicity, consider the case of one input variable A and two output variables B and C. Suppose that the experimenter prepares N copies of the maximally entangled state and sends half of each entangled state through one instance of the unknown process. With this choice, the output state consists of N copies of the state Σ_x, $x \in \{1, 2\}$, with

$$\Sigma_1 = \left[(1-p)\Phi + p\frac{I\otimes I}{d^2}\right]_{BR} \otimes \left(\frac{I}{d}\right)_C \quad \text{and} \quad \Sigma_2 = \left(\frac{I}{d}\right)_B \otimes \left[(1-p)\Phi + p\frac{I\otimes I}{d^2}\right]_{CR}, \tag{126}$$

where Φ is the projector on the canonical maximally entangled state. Then, the quantum Chernoff bound [12] yields the rate

$$\begin{aligned} R &= -\log \min_{0\le s\le 1} \mathrm{Tr}[\Sigma_1^s \Sigma_2^{1-s}] \\ &= -\log \min_{0\le s\le 1} \frac{1}{d^2}\left[\left(1-p+\frac{p}{d^2}\right)^s + (d^2-1)\left(\frac{p}{d^2}\right)^s\right]\left[\left(1-p+\frac{p}{d^2}\right)^{1-s} + (d^2-1)\left(\frac{p}{d^2}\right)^{1-s}\right] \\ &= 2\log d - 2\log\left[\sqrt{1-p+\frac{p}{d^2}} + \sqrt{\frac{p}{d^2}}\right]. \end{aligned} \tag{127}$$

When p is small enough, the rate can be larger than $\log d$, the best classical rate in the noiseless scenario. Since noise can only increase the error probability, this implies a quantum-over-classical advantage in the noisy scenario. The same result holds for the discrimination of $k \geq 2$ hypotheses, as the quantum Chernoff bound for multiple states is equal to the worst-case Chernoff bound among all pairs [8].

We now provide a partial discussion of the scenario where the functional dependence between cause and effect is unknown. This scenario can be modelled by concatenating the depolarizing channel with a completely unknown unitary gate acting on the input variable. The full analysis of the probability of error is substantially more complex, and we leave it as a topic of future research. Here we evaluate the error probability in the simplified scenario where the depolarization is heralded, meaning that when the system is depolarized to the maximally mixed state, the process outputs a classical outcome. Taking this piece of information into account, the error probability becomes $p_{\rm err} = \sum_{k=0}^{N} (1-p)^k \, p^{N-k} \binom{N}{k} \, p_{{\rm err},k}$, where $p_{{\rm err},k}$ is the probability of error with k noiseless experiments.

The evaluation of $p_{{\rm err},k}$ is as follows. The input state of k maximally entangled states, averaged over all possible unitary gates is

$$\rho_{\rm in} = \bigoplus_{\lambda} p_\lambda \, \frac{P_\lambda \otimes P_\lambda}{d_\lambda^2} \otimes \Phi_\lambda \,, \tag{128}$$

where $p_\lambda = d_\lambda m_\lambda / d^N$ is the Schur-Weyl measure, and Φ_λ is the maximally entangled state in $\mathcal{M}_\lambda \otimes \mathcal{M}_\lambda$.

The two output states corresponding to the two hypotheses are

$$\rho_{\rm out,1} = (\rho_{\rm in})_{\mathbf{BR}} \otimes \left(\frac{I}{d}\right)^{\otimes k}_{\mathbf{C}} \qquad \text{and} \qquad \rho_{\rm out,2} = \left(\frac{I}{d}\right)^{\otimes k}_{\mathbf{B}} \otimes (\rho_{\rm in})_{\mathbf{CR}} \,. \tag{129}$$

The distance between them is

$$\begin{aligned}
\|\rho_{\rm out,1} - \rho_{\rm out,2}\|_1 &= \left\| \bigoplus_{\lambda,\mu,\nu} \frac{(P_\lambda)_{\mathbf{B}} \otimes (P_\mu)_{\mathbf{R}} \otimes (P_\nu)_{\mathbf{C}}}{d_\lambda d_\mu d_\nu} \otimes \left[p_\lambda p_\nu \delta_{\lambda\mu} (\Phi_\lambda)_{\mathbf{BR}} \otimes \left(\frac{Q_\nu}{m_\nu}\right)_{\mathbf{C}} - p_\lambda p_\mu \delta_{\mu\nu} \left(\frac{Q_\lambda}{m_\lambda}\right)_{\mathbf{B}} \otimes (\Phi_\mu)_{\mathbf{RC}} \right] \right\|_1 \\
&= 2\left(1 - \sum_\lambda p_\lambda^2\right) + \sum_\lambda p_\lambda^2 \left\| (\Phi_\lambda)_{\mathbf{BR}} \otimes \left(\frac{Q_\lambda}{m_\lambda}\right)_{\mathbf{C}} - \left(\frac{Q_\lambda}{m_\lambda}\right)_{\mathbf{B}} \otimes (\Phi_\lambda)_{\mathbf{CR}} \right\|_1 \\
&= 2\left(1 - \sum_\lambda p_\lambda^2\right) + 2\sum_\lambda p_\lambda^2 \sqrt{1 - m_\lambda^{-2}} \,,
\end{aligned}$$

where the second term in the sum has been evaluated through Equation (42).

Hence, we have the approximate expression

$$\begin{aligned}
\|\rho_{\rm out,1} - \rho_{\rm out,2}\|_1 &= 2\left(1 - \sum_\lambda p_\lambda^2\right) + 2\sum_\lambda p_\lambda^2 \left[1 - \frac{1}{2m_\lambda^2} + O(m_\lambda^4)\right] \\
&= 2 - \frac{1}{d^{2k}} \sum_\lambda d_\lambda^2 + O(d^{-4k}) \\
&= 2 - \frac{{\rm Poly}(k,d)}{d^{2k}} + O(d^{-4k}) \,,
\end{aligned} \tag{130}$$

having used the fact that the dimensions and the number of Young diagrams grow at most polynomially in k and d (see e.g. [10, 11]). Using the above expression, we obtain the expression $p_{{\rm err},k} = \frac{{\rm Poly}(k,d)}{d^{2k}} + O(d^{-4k})$. Summing over k and averaging with the Bernoulli distribution we finally obtain $p_{\rm err} = {\rm Poly}(N,d)\left(\frac{1-p}{d^2} + p\right)^N$ at the leading order.

In conclusion, the discrimination rate is $R = -\log\left(\frac{1-p}{d^2} + p\right)$, which is larger than the noiseless classical rate $\log d$ when p is smaller than $1/(d+1)$. The rate $R = -\log\left(\frac{1-p}{d^2} + p\right)$ provides an upper bound to the achievable rate without heralding, for the simple strategy consisting in preparing N copies of the maximally entangled state. When the probability of depolarisation exceeds $1/(d+1)$ this simple quantum strategy cannot beat the noiseless classical rate, and comparison between quantum and classical strategies requires a more detailed analysis.

It is worth noting the above derivation provides an alternative strategy for the identification of the causal intermediary in the noiseless case ($p = 0$). This strategy achieves the same rate of our universal strategy, although with a polynomially worse error probability. While suboptimal, the present strategy is practically interesting because it does not require input states with large-scale multipartite entanglement.

A. Supplementary Note 8: Proof of Equation (29) in the main text

Step 1. Observe that the channels $\mathcal{C}_\pm = \frac{2}{d^N \pm 1} P_\pm \left(\rho \otimes I^{\otimes N}\right) P_\pm$ are no-signalling. Indeed, for every subset $\mathsf{S} \subseteq \{1, \dots, N\}$ one has that the input system $\mathbf{A}(S) := \bigotimes_{i\in\mathsf{S}} A_i$ cannot signal to the output system $\mathbf{BC}(\overline{\mathsf{S}}) := \bigotimes_{i\notin\mathsf{S}}(B_i \otimes C_i)$. To check the no-signalling condition, we use the relation

$$P_\pm = \frac{I \pm \mathrm{SWAP}}{2} = \frac{\left(\bigotimes_{i\in\mathsf{S}} I_{B_iC_i}\right) \otimes \left(\bigotimes_{i\notin\mathsf{S}} I_{B_iC_i}\right) \pm \left(\bigotimes_{i\in\mathsf{S}} \mathrm{SWAP}_{B_iC_i}\right) \otimes \left(\bigotimes_{i\notin\mathsf{S}} \mathrm{SWAP}_{B_iC_i}\right)}{2}, \tag{131}$$

where $I_{B_iC_i}$ is the identity operator on the composite system B_iC_i, and $\mathrm{SWAP}_{B_iC_i}$ is the unitary operator that swaps systems B_i and C_i. The state of the output system $\mathbf{BC}(\overline{\mathsf{S}})$ is

$$\begin{aligned}\left(\bigotimes_{i\in\mathsf{S}} \mathrm{Tr}_{B_iC_i}\right)[\mathcal{C}_\pm(\rho)] \propto d^{|\mathsf{S}|}\left(\bigotimes_{i\in\mathsf{S}} \mathrm{Tr}_{B_i}\right)\Big[\rho\Big] \otimes \left(\bigotimes_{i\notin\mathsf{S}} I_{C_i}\right) + d^{|\mathsf{S}|}\left(\bigotimes_{i\notin\mathsf{S}} I_{C_i}\right) \otimes \left(\bigotimes_{i\in\mathsf{S}} \mathrm{Tr}_{B_i}\right)\Big[\rho\Big] \\ \pm \left[\left(\bigotimes_{i\in\mathsf{S}} \mathrm{Tr}_{B_i}\right)\Big[\rho\Big] \otimes \left(\bigotimes_{i\notin\mathsf{S}} I_{C_i}\right)\right]\left(\bigotimes_{i\notin\mathsf{S}} \mathrm{SWAP}_{B_iC_i}\right) \\ \pm \left(\bigotimes_{i\notin\mathsf{S}} \mathrm{SWAP}_{B_iC_i}\right)\left[\left(\bigotimes_{i\in\mathsf{S}} \mathrm{Tr}_{B_i}\right)\Big[\rho\Big] \otimes \left(\bigotimes_{i\notin\mathsf{S}} I_{C_i}\right)\right]\end{aligned} \tag{132}$$

and depends only on the state of the input system $\mathbf{A}(\overline{\mathsf{S}})$.

Step 2. Show that there exist coefficients a and b such that the maps $a\,\mathcal{C}_+ + b\,\mathcal{C}_- - 1/2\,\mathcal{C}_{1,I}$ and $a\,\mathcal{C}_+ + b\,\mathcal{C}_- - 1/2\,\mathcal{C}_{2,I}$ are completely positive.

Let us consider the $N = 1$ case first. By definition, one has

$$a\,\mathcal{C}_+ + b\,\mathcal{C}_- - 1/2\,\mathcal{C}_{1,I} = \mathcal{M} \circ (\mathcal{I} \otimes I) \qquad \text{and} \qquad a\,\mathcal{C}_+ + b\,\mathcal{C}_- - 1/2\,\mathcal{C}_{1,I} = \mathcal{M} \circ (I \otimes \mathcal{I}), \tag{133}$$

where $\mathcal{M}$ is the linear map defined by

$$\mathcal{M}(A) := m_{00}\, A + m_{01}\, A\,(\mathrm{SWAP}) + m_{10}\,(\mathrm{SWAP})\, A + m_{11}\,(\mathrm{SWAP})\, A\,(\mathrm{SWAP}) \tag{134}$$

with

$$\begin{aligned} m_{00} &= \frac{a}{2(d+1)} + \frac{b}{2(d-1)} - \frac{1}{2d} \qquad & m_{10} &= \frac{a}{2(d+1)} - \frac{b}{2(d-1)} \\ m_{10} &= \frac{a}{2(d+1)} - \frac{b}{2(d-1)} \qquad & m_{11} &= \frac{a}{2(d+1)} + \frac{b}{2(d-1)}\,. \end{aligned} \tag{135}$$

Now, if the matrix M is positive, then the map $\mathcal{M}$ is completely positive. Defining

$$\alpha := \frac{a}{2(d+1)} \qquad \text{and} \qquad \beta := \frac{b}{2(d-1)}, \tag{136}$$

the positivity condition becomes

$$\alpha + \beta \geq 1/(2d) \tag{137}$$

$$4\alpha\beta \geq (\alpha + \beta)/(2d)\,. \tag{138}$$

As an ansatz, we choose $\alpha = \sqrt{d-1}\,x$ and $\beta = \sqrt{d+1}\,x$, for some $x > 0$. Then, condition (138) becomes

$$x \geq \frac{1}{8d}\left(\frac{1}{\sqrt{d+1}} + \frac{1}{\sqrt{d-1}}\right) =: x_0\,. \tag{139}$$

Note that the choice $x = x_0$ satisfies both conditions (138) and (137). Finally, note that the above derivation holds for arbitrary N, by replacing d with d^N.

Step 3. Define the constant $\lambda := a + b$ and the no-signalling channel $\mathcal{C} := (a\,\mathcal{C}_+ + b\,\mathcal{C}_-)/\lambda$. By construction, the maps $\lambda\,\mathcal{C} - 1/2\,\mathcal{C}_{1,I}$ and $\lambda\,\mathcal{C} - 1/2\,\mathcal{C}_{2,I}$ are completely positive. Explicit evaluation yields

$$\lambda = \frac{\left(\sqrt{d^N+1} + \sqrt{d^N-1}\right)^2}{4d^N}\,. \tag{140}$$

Finally, observe that the maps $\lambda\,\mathcal{C} - 1/2\,\mathcal{C}_{1,I}$ and $\lambda\,\mathcal{C} - 1/2\,\mathcal{C}_{2,I}$ are completely positive if and only if the Choi operators $C, C_{1,I}$, and $C_{2,I}$ corresponding to $\mathcal{C}, \mathcal{C}_{1,I}$, and $\mathcal{C}_{2,I}$ satisfy the inequalities $\lambda\, C \geq 1/2\, C_{1,I}$ and $\lambda\, C \geq 1/2\, C_{2,I}$. Inserting the expression of λ into Equation (26) of the main text, we then obtain the desired bound

$$p_{\mathrm{err}}^{\mathrm{ind}} \geq 1 - \lambda = \frac{1 - \sqrt{1 - \frac{1}{d^{2N}}}}{2}\,. \tag{141}$$

[1] Chiribella, G., D'Ariano, G. M. & Roetteler, M. Identification of a reversible quantum gate: assessing the resources. *New Journal of Physics* **15**, 103019 (2013).

[2] Helstrom, C. W. Quantum detection and estimation theory. *Journal of Statistical Physics* **1**, 231–252 (1969).

[3] Hausladen, P. & Wootters, W. K. A pretty good measurement for distinguishing quantum states. *Journal of Modern Optics* **41**, 2385–2390 (1994).

[4] Yuen, H., Kennedy, R. & Lax, M. Optimum testing of multiple hypotheses in quantum detection theory. *IEEE Transactions on Information Theory* **21**, 125–134 (1975).

[5] Holevo, A. S. *Probabilistic and statistical aspects of quantum theory*, vol. 1 (Springer Science & Business Media, 2011).

[6] Chiribella, G. Group theoretic structures in the estimation of an unknown unitary transformation. In *Journal of Physics: Conference Series*, vol. 284, 012001 (IOP Publishing, 2011).

[7] Fulton, W. & Harris, J. *Representation theory: a first course*, vol. 129 (Springer Science & Business Media, 2013).

[8] Li, K. *et al.* Second-order asymptotics for quantum hypothesis testing. *The Annals of Statistics* **42**, 171–189 (2014).

[9] Mozrzymas, M., Studziński, M., Strelchuk, S. & Horodecki, M. Optimal port-based teleportation. *New Journal of Physics* **20**, 053006 (2018).

[10] Harrow, A. W. Applications of coherent classical communication and the schur transform to quantum information theory. *Preprint at https://arxiv.org/abs/quant-ph/0512255* (2005).

[11] Christandl, M. & Mitchison, G. The spectra of quantum states and the kronecker coefficients of the symmetric group. *Communications in mathematical physics* **261**, 789–797 (2006).

[12] Audenaert, K. M. *et al.* Discriminating states: The quantum chernoff bound. *Physical review letters* **98**, 160501 (2007).
